# Complement Fragment C3a Controls Mutual Cell Attraction during Collective Cell Migration

**DOI:** 10.1016/j.devcel.2011.10.012

**Published:** 2011-12-13

**Authors:** Carlos Carmona-Fontaine, Eric Theveneau, Apostolia Tzekou, Masazumi Tada, Mae Woods, Karen M. Page, Maddy Parsons, John D. Lambris, Roberto Mayor

**Affiliations:** 1 Department of Cell and Developmental Biology, University College London, Gower Street, London WC1E 6BT, UK; 2 Department of Mathematics and CoMPLEX, University College London, Gower Street, London WC1E 6BT, UK; 3 Department of Pathology & Laboratory Medicine, University of Pennsylvania, Philadelphia, PA 19104-6055, USA; 4 Randall Division of Cell and Molecular Biophysics, King's College London, London WC2R 2LS, UK

## Abstract

Collective cell migration is a mode of movement crucial for morphogenesis and cancer metastasis. However, little is known about how migratory cells coordinate collectively. Here we show that mutual cell-cell attraction (named here coattraction) is required to maintain cohesive clusters of migrating mesenchymal cells. Coattraction can counterbalance the natural tendency of cells to disperse via mechanisms such as contact inhibition and epithelial-to-mesenchymal transition. Neural crest cells are coattracted via the complement fragment C3a and its receptor C3aR, revealing an unexpected role of complement proteins in early vertebrate development. Loss of coattraction disrupts collective and coordinated movements of these cells. We propose that coattraction and contact inhibition act in concert to allow cell collectives to self-organize and respond efficiently to external signals, such as chemoattractants and repellents.

## Graphical abstract

**Graphical Abstract:**

**Figure:**
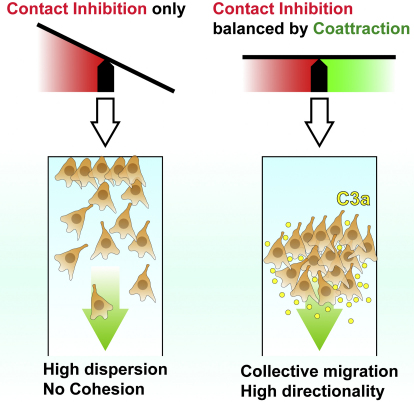

**Highlights:**

► Neural crest (NC) cells mutually attract each other (coattraction) ► NC coattraction is mediated by the complement fragment C3a and its receptor C3aR ► Coattraction is required for the swarm-like behavior of migratory NC cells ► Coattraction and contact inhibition suffice to generate collective migration

## Introduction

During collective migration, cells must coordinate to achieve cohesive and coherent movement. This type of migration is widely used by embryonic tissues and during metastasis. Despite its importance for health and disease (Friedl and Gilmour, 2009; Friedl and Wolf, 2003; Montell, 2008; Rørth, 2009; Wolf et al., 2007), little is known about how these cells coordinate. During cancer progression, malignant cells usually undergo epithelial to mesenchymal transition (EMT), a process where cell-cell adhesion is greatly reduced, before metastasis (Thiery et al., 2009). Intriguingly, these mesenchymal cells can migrate in a collective fashion (Friedl and Gilmour, 2009; Friedl and Wolf, 2003), suggesting that cohesive mechanisms, other than cell adhesion, may exist in collective migration of mesenchymal cells. To address this, we have analyzed the collective migration of *Xenopus* and zebrafish neural crest (NC) cells, a multipotent embryonic cell population that undergoes EMT before acquiring invasive migratory properties reminiscent of malignant cells (Carmona-Fontaine et al., 2008; Erickson and Reedy, 1998; Kuriyama and Mayor, 2008; Mayor and Carmona-Fontaine, 2010; Sauka-Spengler and Bronner-Fraser, 2008). Surprisingly, our results show that NC cells spontaneously display collective migration in which cohesion is achieved via a mutual chemoattraction mechanism, named here coattraction. We show that complement factor C3a and its receptor C3aR correspond to the ligand and receptor, coexpressed in NC cells, responsible for coattraction.

## Results

### NC Cells Mutually Attract One Another

**Figure 1:**
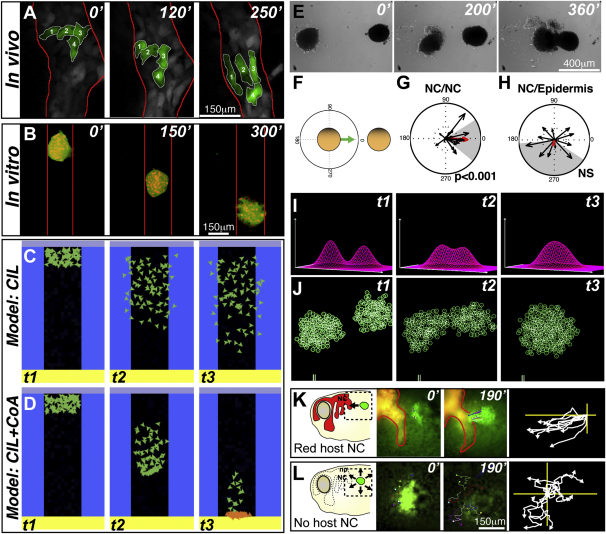
NC Cells Exhibit Mutual Cell Attraction (A) NC migration in vivo in a zebrafish embryo (time in minutes). Colored cells show that they maintain their neighbor relations during migration in vivo. (B) In vitro *Xenopus* migratory NC cell cluster showing spontaneous collective migration in a restricted space. (C and D) Temporal evolution of a computer model that shows that CIL alone leads to cell dispersion (C), but if an attractive interaction (coattraction [CoA]) counterbalances CIL, collective migration may emerge (D). Cells are allowed to migrate in the black zone and repelled by the blue borders. They stop moving when they reach the bottom and turn orange. (E) NC explants attract each other in vitro. (F) Quantification method to determine CoA vector. (G and H) CoA vector plots. NC/NC confrontation pairs show significant CoA (G; p < 0.001; n = 36), but NC/Epidermis pairs do not (H; NS, n = 12). NS, not significant. Red arrow shows average vector. Gray area indicates circular dispersion. (I and J) Temporal evolution of a computational model showing that CoA is feasible. (I) Profiles of the attractant concentration. (J) Spatial distribution of two groups of particles. Note that the two groups move toward each other. (K and L) CoA in vivo. Left panel shows experimental scheme. Middle panels illustrate start and end point. Right panel indicates centered tracks. Grafts of labeled NC cells (green) join endogenous (red) NC cells (K; 77%; n = 13) but disperse radially when grafted in embryos without NC cells (L; 0%; n = 15). See also Figure S1 and Movies S1, S2, and S3.

Despite being a mesenchymal cell population, migratory zebrafish NC cells display a high degree of coherence in vivo; all cells move with high persistence and maintain the same neighbors for long periods of time via transient and dynamic contacts (Figure 1A) (Carmona-Fontaine et al., 2008; Teddy and Kulesa, 2004). Intriguingly, when cultured in vitro, *Xenopus* NC cells can self-organize to adopt spontaneous collective migration highlighting the role of local interactions and suggesting that no external cues are required for this organization (Figure 1B; see Movie S1 available online). Contact inhibition of locomotion (CIL), a repulsive local interaction required for NC directional migration (Carmona-Fontaine et al., 2008), is predicted to result in cell dispersion (Mayor and Carmona-Fontaine, 2010) that does not fit with the observed spontaneous cohesive movement. Given cell adhesion in these cells is diminished (Nakagawa and Takeichi, 1995, 1998; Theveneau et al., 2010), we hypothesize that to maintain a cluster configuration, an attractive interaction between NC cells might be required to counterbalance CIL-dependent repulsion. To test the plausibility of this idea, we created an agent-based model of NC migration where different cell-cell interactions were taken into account (see “Computer model of Neural Crest migration” in Supplemental Experimental Procedures). First, randomly moving particles were giving a repulsive interaction similar to CIL (Figure 1C). This interaction greatly enhanced the efficiency of migration with respect to noninteracting cells (Movie S2) but failed to reproduce the cohesive movement we have just described. However, if in addition to CIL an attractive interaction between cells is added, these particles display efficient and cohesive collective migration (Figure 1D). We have coined the term coattraction for this putative interaction. The qualitative behavior of this model is very robust because it does not depend on the specific values of its parameters. Hence, this model is consistent with the proposal that a combination of CIL and coattraction can suffice for the emergence of collective migration. Interestingly, similar models of collective movements of organisms ranging from bacteria to animals (Buhl et al., 2006) show that repulsive (like CIL) plus attractive interactions (like coattraction) suffice to generate swarming behavior (Romanczuk et al., 2009), i.e., collective migration.

Observation of NC migration in vivo revealed that when a NC cell departs from its migratory stream, it always returns (Figures S1A and S1B) (Teddy and Kulesa, 2004), and NC cells in vitro frequently do so as well (Figure S1C). This observation further supports the idea that NC cells can mutually attract each other. To directly test coattraction, we studied the behavior of NC explants, clusters consisting in a few hundred of NC cells. When cultured alone, clusters disperse radially or displace randomly. However, a completely different behavior is observed when two early migratory NC explants are cultured in close proximity but distant enough to rule out any kind of physical contact (∼500 μm, i.e., more than ten cell diameters). In this situation the two groups of cells consistently move toward each other (Figure 1E; Movie S3). Quantification of this behavior (Figures 1F and 1G) shows that directional bias is highly significant when NC explants are confronted to other NC explants, but not when they are confronted to epidermal explants (Figures 1G and 1H). This surprising observation demonstrates that NC cells exhibit coattraction because it is tissue specific, nonrandom, and there is no physical contact between the confronted explants. Importantly, in a similar model to the one shown before, but where particles were disposed as two confronted groups, we managed to demonstrate that two populations that produce and sense an attractant join each other (Figures 1I and 1J). A physical intuition of the effect of a cell cluster on another one can be gained by considering that two opposite forces work on each cell: CIL that leads to cells moving away from the cluster, and coattraction that produces an inward movement of cells (see implementation of the model in Supplemental Experimental Procedures). The presence of a second cluster decreases the gradient of coattractant in the direction of the second group (compare gradients between clusters in Figure 1I at t1 and t2) and in consequence, reduces coattraction in that direction, which leads to the outward movement of cells from the first group in the direction of the second cluster. The same process takes place in the second cluster with the final outcome being that both groups of cells move toward each other.

To test coattraction in vivo, labeled NC explants were grafted near the NC of another embryo. These grafts migrate directionally to join the endogenous NC streams (Figure 1K; Movie S3). However, similar NC grafts disperse radially when grafted onto embryos whose NC had previously been removed (Figure 1L; Movie S3). It is interesting to mention that the maximal distance at which coattraction works in vivo is around 100 μm, which is smaller than the normal distance between NC streams. This observation, together with the fact that repellent molecules are expressed between the streams (Davy et al., 2004; Eickholt et al., 1999; Gammill et al., 2006), explains why there is no coattraction between the streams. Altogether, these results demonstrate that NC cells attract each other via coattraction.

### C3a and Its Receptor C3aR Are Required for NC Migration

**Figure 2:**
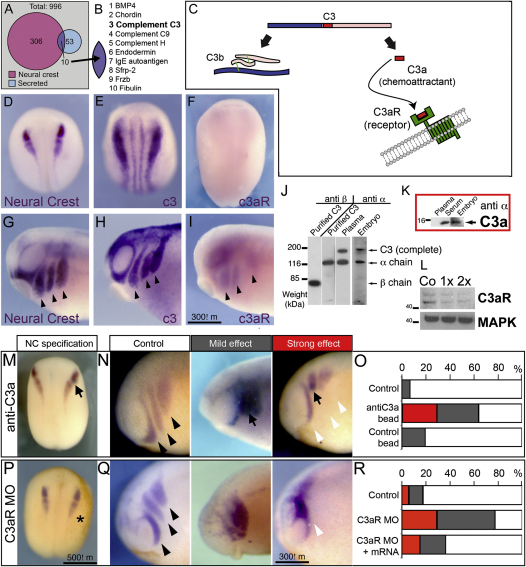
C3a and C3aR Are a Chemotactic Pair, Both Produced and Sensed by NC Cells and Required for NC Migration (A) Venn diagram of genes expressed in the NC (pink) and that encode secreted proteins (light blue), from a total of 996 genes (gray) whose expression patterns during *Xenopus* development have been previously determined (Pollet et al., 2005). (B and C) Ten candidate genes were found (purple) (B). From these genes C3 was particularly interesting because when it is cleaved, it releases a chemotactic peptide C3a that binds to its receptor C3aR (C). (D–F) Premigratory NC stages. (D) Expression of NC markers *snail2* and *twist*. (E) C3 expression in the NC. (F) C3aR in situ hybridization showing no NC expression. (G–I) Migratory NC stages. Arrowheads indicate migratory streams. (G) Expression of NC markers *snail2* and *twist*. (H) C3 expression. (I) C3aR expression. (J and K) C3 proteins. C3 (J) and C3a (K) are detected by western blot. Lanes are loaded with purified proteins or *Xenopus* extracts as indicated. Anti-α and anti-β are specific antibodies for corresponding C3 chains. (L) C3aR western blots of *Xenopus* extract injected with a control MO (Co) or different doses (1×, 2×) of C3aR MO. Note the decrease in C3aR protein by the C3aR MO. (M–R) Analysis of cell migration via in situ hybridization. Embryos treated with a bead soaked in antiC3a (black arrow) to inhibit C3a (M–O) or with C3aR MO (asterisk) to block C3aR (P–R) were fixed at NC specification (M and P) or migration stages (N and Q). Then, NC specification and migration were assessed using the expression of *snail* and *twist*, genes specifically expressed in NC cells. (M) C3a inhibition does not affect NC specification. (N) Range of effect in NC migration after C3a inhibition. (O) Quantification of the phenotypes as percentage of the total. Note the specificity of the treatment because beads coated with control antibodies do not significantly affect NC migration (n > 20 for all cases). (P–R) Similar to (A)–(C) but after treatment with C3aR MO. Note the rescue of C3aR mRNA showing the specificity of C3aR MO (R). See also Figure S2.

To elucidate the molecular mechanism of NC coattraction, we searched for genes encoding secreted proteins expressed in the NC using in situ hybridization databases (Pollet et al., 2005) (Figure 2A). This revealed ten candidates (Figure 2B), among which was C3, a central component of the complement pathway (Figure 2C). C3 is cleaved to produce C3a, a small anaphylatoxin peptide with known chemotactic properties in the immune system (Ricklin et al., 2010), and thus, a good candidate to be the chemoattractant required for coattraction. We found that C3 is expressed in NC cells (Figures 2D, 2E, 2G, and 2H), and importantly, we found that the receptor *c3aR* (Figures S2A–S2E) is also expressed in migratory but not in premigratory NC cells (Figures 2F and 2I). Furthermore, C3 (including C3a) and C3aR proteins are present in migrating NC cells (Figures 2J–2L; Figure S2A). Thus, migrating NC cells produce both the ligand C3a and its receptor, C3aR.

To study the role of C3a/C3aR in NC migration, we generated blocking antibodies for C3a and C3aR (Supplemental Experimental Procedures), and we designed a morpholino to block the translation of C3aR (efficiency analyzed by western blot in Figure 2L). To inhibit C3a, beads soaked in C3a antibody were grafted next to the NC, and to inhibit C3aR, a C3aR MO was injected into one blastomere of an eight cell stage embryo. Inhibition of C3a and C3aR in the NC produces very similar behaviors: loss of NC migration resulting in phenotypes that ranges in severity from disruption of NC streams to complete disorganization of NC migration with seldom net displacement (Figures 2N, 2O, 2Q, and 2R). Graft of PBS or IgG beads did not affect NC migration (Figure 2O), indicating the specificity of the antibody treatment. The specificity of the C3aR MO was shown by rescuing NC migration by coinjection of C3aR MO and a non-MO-binding C3aR mRNA (Figure 2R). Importantly, inhibition of C3a and C3aR does not affect NC formation (Figures 2M and 2P). In conclusion, C3a and its receptor C3aR are expressed in NC cells and are required for their migration.

### C3a Is an NC Chemoattractant

**Figure 3:**
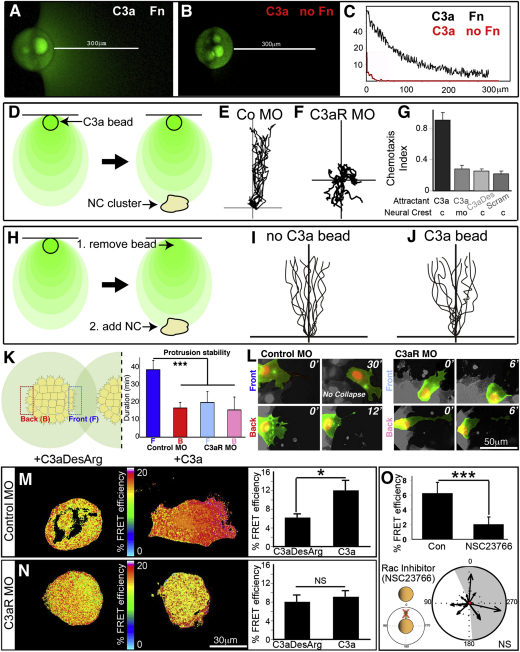
C3a Works as a Neural Crest Chemoattractant (A–E) C3a forms a stable gradient on fibronectin. Under saline medium, beads (C3a-coated or not) were placed on silicone grease on a surface with or without fibronectin. After 1–3 hr, the surface was carefully washed and then C3a was detected using immunofluorescence. Note that no signal was observed in the absence of C3a or of the C3a primary antibody (not shown). (A) C3a forms a gradient on fibronectin. (B) Similar to (A), but the C3a-soaked bead was placed on a surface without fibronectin. Note that no C3a is detected outside the bead region. (C) Quantification of fluorescence level for ten different beads. The x axis shows distance to the bead in micrometers (μm), and the y axis indicates fluorescent intensity, in arbitrary units. (D–J) C3a is a NC chemoattractant. (D) Experimental design. C3a beads were fixed on fibronectin, and NC cells were cultured next to the bead. (E) Tracks of control MO NC clusters exposed to a C3a source (chemotaxis index [CI], 0.987; p < 0.001). (F) Tracks of C3aR MO NC clusters exposed to a C3a source (CI: 0.211; not significant). (G) Summary of the CI for different treatments. Control (c) or C3aR morphant (mo) NC cells were used. At least three experiments for each condition were performed. (H–J) Stability of the C3a gradient. (H) Experimental design. A C3a bead was placed on a fibronectin surface for 1–3 hr, which suffice to establish a gradient (A and C). Next, the bead was removed, and a NC cluster was placed next to the position where the bead was. Then, time-lapse and tracking of migrating cells were performed (I). As a control, tracks of NC cells where the C3a bead was not removed are shown (J). No difference in NC chemotaxis was observed between migration to C3a-coated beads and through the C3a bound to fibronectin only. (K and L) Confronted explants stabilize protrusions in a C3a-dependent manner. (K) Stability of cell protrusions was measured and compared between cells at the front and back, as shown in the cartoon on the left. Note that the difference between front and back cells is lost when C3aR is blocked (^∗∗∗^p < 0.001). (L) Representative images of recently formed protrusions and the time when they collapsed under the different conditions. Note that control front protrusions remain stable for more than 30 min. (M–O) C3a regulates Rac activity. (M and N) Rac1 activity analyzed by FRET. (M) Representative cases of control and c3a-treated cells are shown. Note the polarized Rac1 activity in the C3a condition. Percentage (%) of FRET efficiency (p < 0.05; control n = 19, C3a n = 17). Note that this method may alter cell morphology. (N) C3aR morphant cells were treated with a control peptide (C3aDesArg) or C3a. No difference in FRET efficiency was observed, indicating that the response to C3a is C3aR dependent. (O) Rac activity analyzed by FRET after treating NC cells with the Rac inhibitor NSC23766, showing an efficient Rac inhibition. Vector plot showing loss of coattraction after Rac1 inhibition. Error bars in (G) and (M)–(O) correspond to the standard deviation from the mean. See also Movie S4.

To study the role of C3a/C3aR in NC migration, we synthesized a C3a agonist together with two control peptides (C3aDesArg, which does not bind the receptor, and scrambled C3a) (Supplemental Experimental Procedures). To test if C3a is a chemoattractant for NC cells, a chemotaxis assay was used (Theveneau et al., 2010); briefly, heparin beads soaked with C3a were placed near NC explants. Importantly, we were able to demonstrate that under these conditions C3a forms a stable gradient by binding to the fibronectin substrate (Figures 3A–3C). Notably, most NC explants showed a strong directional bias toward the C3a source indicating chemotaxis (Figures 3D, 3E, and 3G; Movie S4). This chemotactic behavior is abolished when C3aR is blocked, showing that NC cells sense C3a via C3aR (Figures 3F and 3G). To test the stability of the C3a gradient and the capacity of NC cells to respond to C3a bound to fibronectin, the C3a beads were removed before adding the NC cells (Figure 3H). Remarkably, after the C3a gradient is formed, no significant difference was observed in chemotaxis when the C3a bead was absent (Figure 3I) or present (Figure 3J), showing that C3a bound to the substrate is sufficient to attract NC cells. Importantly, none of the control (scrambled or C3aDesArg) peptides was able to attract NC cells, demonstrating that the chemotactic effect of C3a is specific (Figure 3G; Movie S4). Together, these experiments show that C3a is a NC chemoattractant and that it works via C3aR.

It has been shown that response to a chemoattractant such as SDF1 leads to stabilization of cell protrusions (Theveneau et al., 2010). We analyzed cell protrusion stability as a readout of chemotaxis response during coattraction between two explants cultured at same distance (∼500 μm). We observed that protrusions of frontal cells (defined as the ones directly opposed to the other explant, Figures 3K and 3L) had similar shape to protrusions at the back but were far more stable (Figures 3K and 3L, graph). Importantly, this difference was lost when C3aR was blocked. These results show that the presence of a neighboring NC cluster stabilizes cell protrusions in the direction of the neighbor cluster and in a C3a-dependent manner. This simple mechanism can bias the collective movement of one explant toward the other one. To further analyze the mechanism by which C3a promotes NC coattraction, we studied the participation of Rho small GTPases in this process. Rac1, a Rho GTPase essential for lamellipodia formation and maintenance (Ridley et al., 1992), has been shown to be required for NC migration and chemotaxis (Matthews et al., 2008; Theveneau et al., 2010). Using FRET, we determined that C3a activates Rac1 in a C3aR-dependent manner (Figures 3M and 3N). Moreover, when Rac1 is inhibited, NC explants lose their coattraction (Figure 3O), supporting the idea that this mechanism occurs by mutual chemoattraction, possibly via Rac1 activated by C3aR upon binding to C3a.

### C3a/C3aR Are Required for NC Coattraction

**Figure 4:**
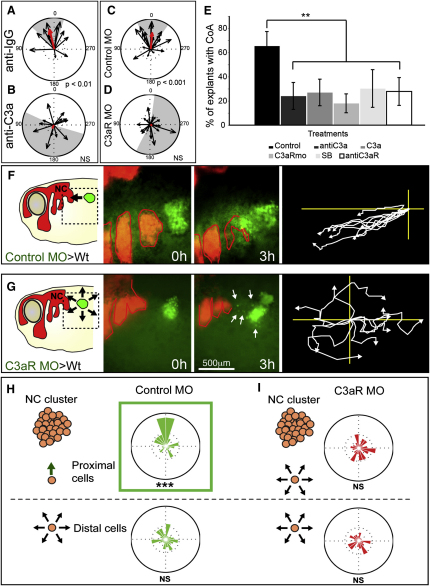
C3a and C3aR Mediate Coattraction (A–D) Vector plots showing that CoA in control IgG (A) is inhibited by a C3a via blocking antibody (B); and that normal CoA in control MO (C) is inhibited by C3aR MO (D). (E) Effect of different C3a/C3aR inhibitors on CoA (p < 0.01; n ∼20 for each experiment). The control bar includes measurements of control peptide (to control the effect of C3a), Rabbit-IgG (to control C3a and C3aR antibodies), control morpholino (to control C3aR MO), and DMSO (to control SB290157). No significant difference was found between different control treatments. Standard deviation obtained from three independent experiments. ^∗∗^p < 0.01. Error bars correspond to the standard deviation from the mean. (F and G) Loss of CoA in vivo. Left panel shows experiment scheme. Middle panels illustrate start and end point. Right panel indicates centered tracks. White arrows show dispersed single cells. (F) Grafts of control NC cells (green) join endogenous (red) NC cells (80%; n = 10). (G) Grafts of C3aR MO cells disperse radially (8%; n = 12). (H and I) Control or C3aR MO NC cells were dissociated into single cells and then scattered in the vicinities of differently labeled NC explants. Rose plots (green, control cells; red, C3aR MO cells). (H) Control cells proximal to the cluster (<300 μm) have a trend to migrate toward the cluster, whereas distant cells are not affected, and they move randomly. (I) Similar experiments for C3aR morphant NC cells show that these cells exhibited random migration, regardless of their distance to the explant. See also Figure S3 and Movie S5.

To test if C3a/C3aR chemotaxis is responsible for coattraction, we employed the confrontation assay described in Figures 1E–1H, in explants treated with different C3a or C3aR inhibitors. Explants were treated with antibodies against C3a and C3aR, with a specific C3a antagonist, SB290157, and cell injected with C3aR MO. All treatments that inhibit either C3a or C3aR impair coattraction (Figures 4A–4E; Movie S5). Similarly, grafts of NC cells lose their ability to join endogenous NC cells when C3aR is inhibited in vivo by a C3aR MO (Figures 4F and 4G; Movie S5), showing that coattraction in vivo also requires C3a/C3aR.

**Figure 5:**
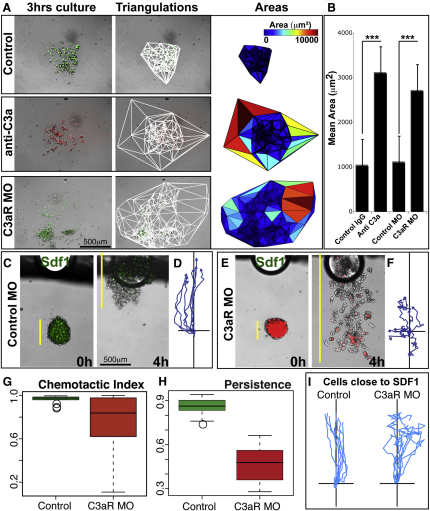
C3a/C3aR Loss of Function Leads to Increased NC Dispersion and a Concomitant Poor Response to External Signals (A) Delaunay triangulations (center) of NC explants after 3 hr of culture (left) show enhanced dispersion after C3a/C3aR loss of function as areas in between neighbors are increased (right). (B) Quantification of many explants (triplicates with n > 10, each) shows that this effect is consistent and significant (^∗∗∗^p < 0.001). (C–I) Response toward Sdf1. Control NC cells respond uniformly to an Sdf1 source (C), whereas C3aR MO cells respond heterogeneously (E). (D and F) Tracks represent the displacement of rear cells. Box plot showing the distribution of chemotaxis indexes (G) and persistence (H). Note that some C3aR cells respond as good as control cells, but they are much more heterogeneous as a group. (I) Tracks for control and C3aR MO cells near the Sdf1 source, showing that both cells are able to sense Sdf1. Error bars in (B), (G), and (H) correspond to the standard deviation from the mean. See also Movie S6.

**Figure 6:**
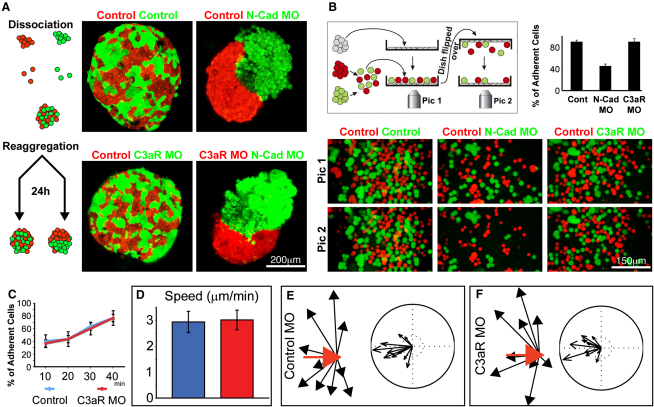
C3a/C3aR Do Not Have a Major Effect on Cell Adhesion, Motility, or CIL (A) Cell-sorting assay to estimate differential cell-cell adhesive properties. Dissociated cells were reaggregated and cultured for 24 hr before analysis. As expected, NC cells labeled with two different colors mix with each other because they show equal adhesive properties. As a positive control for cell sorting, control NC cells were mixed with N-Cadherin morphant NC cells. Clear cell segregation is observed. However, C3aR MO does not affect cell sorting of NC cells because morphant cells mix perfectly with control and segregate from N-Cad MO NC cells. (B) Cell-cell adhesion assay. NC cells were cultured as a monolayer on fibronectin (gray cells). Control (red) and treated (green) NC cells were dissociated, mixed, and seeded on the NC monolayer. After 3–5 min, the dish was flipped over, shaken, and the number of remaining cells was counted. Pictures were taken before (Pic 1) and after (Pic 2) the dish was flipped over. Most of the control cells remained adhered to the NC monolayer; however, a large proportion of the N-Cadherin MO cells was lost, indicating a decrease in NC-NC adhesion in the N-Cadherin depleted cells. C3aR MO cells remained adhered to the NC monolayer, suggesting no major effect on cell-cell adhesion. (C) Quantification of the adhesion to substrate assay. Control (blue) or C3aR MO (red) NC explants were cultured on fibronectin, and the culture dish was flipped over at the indicated times. The percentage of adhered explants was then quantified. Standard deviation was obtained from three independent experiments (p > 0.05). (D) Cell motility is not affected by C3aR MO as single control (blue bar), and C3aR MO (red bar) cells show the same speed of migration. (E and F) Control and C3aR MO NC cells have normal CIL. Confrontation of NC explants to measure CIL was performed as described (Carmona-Fontaine et al., 2008). Red arrows show velocity vector before collision; black arrows indicate velocity vector after collision. Cluster of acceleration vectors is not changed by C3aR MO, indicating that CIL if not affected. Error bars in (B) and (D) correspond to the standard deviation from the mean.

We predicted that coattraction is required for collective NC migration (Figure 1D). In fact, the trend of single cells to return to the cluster observed in control explants is lost when C3aR was blocked. To quantify this effect, control or C3aR MO NC cells were dissociated and then scattered around untreated NC clusters. As expected, these cells move randomly when they are distant from a NC cluster (>300 μm). However, when control but not C3aR MO cells are close to the cluster, they switch to a directional movement, frequently joining the cluster (Figures 4H and 4I; Figure S3). Following the same logic, we hypothesized that C3a or C3aR loss of function should increase the dispersion of NC explants. We devised a method to calculate cell dispersion that is independent of the size of the explant. First, for each cell we determine its two closest neighbors using a Delaunay triangulation algorithm (Supplemental Experimental Procedures). Then, the areas of the formed triangles (which are proportional to cell dispersion) are measured and compared (Figure 5A). As it is shown in Figures 5A and 5B, the inhibition of either C3a or C3aR leads to enhanced dispersion of NC clusters. Importantly, these treatments do not affect cell adhesion (see below) (Figure 6). Hence, these experiments suggest that C3a/C3aR-mediated coattraction is required to maintain a cohesive NC explant.

### C3a/C3aR Are Required for Collective Response to External Chemoattractants

It has recently been shown that NC groups respond better than single cells to extrinsic chemoattractants such as Sdf1 (Theveneau et al., 2010), but how this cluster configuration is maintained remains unclear. We performed a chemotaxis assay toward Sdf1 in cells injected with a control MO (Figure 5C) or with C3aR MO (Figure 5E). As shown by the cell tracks in Figures 5D and 5F (Movie S6), chemotaxis toward Sdf1 was greatly impaired by blocking C3a function. This result cannot be explained by an effect of C3aR MO on the sensitivity to Sdf1 because C3aR-depleted cells placed close to the Sdf1 source respond as control cells (Figure 5I). Instead, when C3aR is blocked, NC cells lose their collective properties and display variable persistence (Figures 5G and 5H). Thus, coattraction within NC cells is required for their collective interpretation of extrinsic signals such as Sdf1.

### C3a/C3aR Do Not Play a Major Role in Cell Adhesion or Motility

All these loss-of-function experiments, of either C3a or C3aR, result in less cohesive NC explants, which we suggest represents a diminished coattraction. However, other alternatives for a role of C3a/C3aR in NC migration are also possible, such as modulation of cell *adhesion*, cell motility, or CIL. In order to test a possible role of C3a/C3aR on cell-cell adhesion, two different experimental approaches were preformed. First, a cell-sorting experiment in which NC cells were dissociated, reaggregated, and cultured for 24 hr shows no difference between untreated and C3aR-deficient cells (Figure 6A). As a positive control, normal NC cells were mixed with N-cadherin morphant cells, showing the expected cell-sorting behavior (Figure 6A). Importantly, C3aR MO does not affect the ability of NC cells to sort out from N-Cad MO cells (Figure 6A). In a second experiment, normal NC cells were cultured as a monolayer, and a mix of control and treated NC cells was deposited over the initial layer (Figure 6B). After a few minutes, the dish was flipped over, shaken, and the remaining cells attached to the monolayer were counted. A larger proportion of cells injected with a morpholino against N-Cadherin was detached from the monolayer compared with control cells, indicating a decrease in cell-cell adhesion, as expected (Figure 6B). However, no difference was observed between control and C3aR morphant cells. Taken together, these experiments suggest that inhibition of C3a/C3aR is not having a major effect on cell-cell adhesion. In order to test for a role of C3a/C3aR on adhesion to fibronectin, NC cells were cultured on this substrate for different times, the dish was flipped over, and the cells remaining in the dish were counted. No difference between control and C3aR cells was observed (Figure 6C). In addition, the speed of migration was compared between control and C3aR morphant cells, and no difference was detected (Figure 6D). Finally, an assay to directly measure CIL (Carmona-Fontaine et al., 2008) was performed. Again, no difference in CIL was observed between normal and C3aR morphant cells (Figures 6E and 6F). Although we cannot completely rule out that C3a/C3aR have undetected effects on cell adhesion, motility, or CIL, they will be minor if any and, thus, unlikely to explain the strong effect in NC migration observed after C3a/C3aR impairment. This reinforces the evidence favoring C3a/C3aR as mediators of coattraction and their crucial role in collective migration.

### C3a/C3aR Control Collective Cell Migration

**Figure 7:**
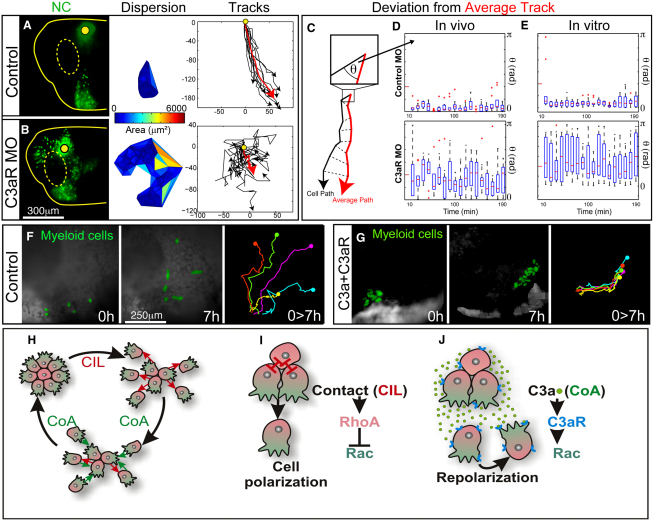
Coattraction Is Required and Sufficient for Collective Cell Migration (A) Analysis of cell migration via live imaging. Cell distribution (left), dispersion (middle), and migratory tracks (right) of control NC cells (green nuclei) after 10 hr of migration in vivo. Yellow lines indicate embryo and eye (dashed) outlines. Red line shows average track. (B) Similar to (A) but for C3aR MO NC cells. (C) Scheme to show the deviation angle from the average direction, θ. (D) Box plot showing the dispersion of θ for each time point. (E) Similar to (D) but for NC migrating in vitro. (F and G) Myeloid cells (green) migrate as individual cells (F) but turn to a more cohesive type of migration if coexpressing C3a and C3aR (G). Left panels show start and end of migration (time: 7 hr). Right panel illustrates representative tracks. (H–J) Model of collective NC migration. (H) CIL leads to cell dispersion, whereas CoA keeps the cells together. A permanent cycle between CIL and CoA is required for collective migration. (I) CIL polarizes NC cells in a contact-dependent manner. This polarization is controlled by localized regulation of small GTPase activities, and allows a more efficient response to external signals. (J) CoA, which is C3a/C3aR dependent, repolarizes cells that are moving away from the cluster and thus keeps cells together. CoA involves activation of Rac and by bringing cells together allows CIL to start again, as depicted in (H). See also Figure S4 and Movies S7 and S8.

The loss-of-function experiments reveal a crucial role for C3a and C3aR in NC migration, without affecting NC formation (Figures 2M–2R). Inhibition of C3a and C3aR in the NC produces embryos with poor migratory NC cells, with little net displacement and fusion of the streams (Figures 2N and 2Q). We hypothesized that this fusion results from NC cells migrating in a disorganized manner and going at random locations. This would differ from a situation where treated streams will fuse by preferentially migrating toward each other. In order to distinguish these possibilities, we performed live imaging and a statistical analysis of in vivo migrating cells after blocking C3a/C3aR signaling. To track the cells, we performed in vivo time-lapse analysis of NC cells expressing nuclear-GFP in embryos injected with control MO (Figure 7A), C3aR MO (Figure 7B), and C3a antibody (Figure S4). As shown in Figure 7A, labeled control NC cells migrate in a cohesive fashion with little dispersion and aligned paths of displacement. In contrast, C3aR morphant cells lose their collective migration and disperse as individuals (Figure 7B; Movie S7). Importantly, this enhanced dispersion does not show any directional bias (Figures 7B–7E), and thus, it does not represent an attraction between streams but a loss in the coherent directionality of these cells after inhibition of coattraction. To quantify the coherence in the movement of these cells, the deviation of each cell from the average path was measured (Figure 7C). Whereas control cells show little angular divergence from the average path, C3aRMO cells show highly divergent displacements and much more variable speeds (Figure 7D; Figures S4A and S4D). To confirm that this is not influenced by cues from other regions of the embryo, we analyzed collective NC migration in vitro, and similar results were obtained (Figure 7E; Figures S4B and S4F–S4K; Movie S7). Altogether, our results show that the C3a/C3aR work as a chemotactic pair that is produced and sensed by NC cells. This role as an intrinsic chemoattractant is responsible for NC coattraction, a key element in its collective migration.

An exciting hypothesis is that coattraction allows the emergence of collective migration in a cell population that would otherwise be dispersed as single cells. To test this, we analyzed the migration of myeloid cells, a cell population that originates at the embryonic anterior ventral blood island from where it disperses as individual cells (Costa et al., 2008), possibly due to CIL (Mayor and Carmona-Fontaine, 2010; Stramer et al., 2010). These cells do not express *c3* or *c3aR* (McLin et al., 2008) (Figures 2D–2I). However, if these cells are engineered to express C3a and C3aR proteins, their typical individual movement (Figure 7F; Movie S8) turns into collective displacement (Figure 7G; Movie S8), indicating that C3a/C3aR-dependent coattraction is sufficient to trigger collective cell migration.

## Discussion

Here we have shown that directional collective migration is a self-organizing property of *Xenopus* and zebrafish NC cells because it does not require, but can better integrate, external signals. We have also shown that coattraction has a crucial role in this process. CIL alone, although essential for NC migration (Carmona-Fontaine et al., 2008), leads to rapid dispersion of the group (Figure 7H, upper part), leaving individual migratory cells that progress poorly as they no longer interact. Thus, coattraction counterbalances this dispersion by maintaining NC cells at a density that allows interactions (Figure 7H, lower part). This density level is required for CIL to maintain the directionality of migration of the cell group (Carmona-Fontaine et al., 2008). Importantly, given that NC cells are mesenchymal cells, with reduced cell adhesion and only transient contacts, it is unlikely that cell adhesion could counterbalance cell dispersion promoted by CIL. However, it is still possible that once NC cells are coattracted and they make new contacts, cell adhesion could play a transient role in maintaining cells together. We propose that coattraction, together with CIL, orchestrates NC cell movements by maintaining a critical cell density that allows them to acquire collective migration and to respond more efficiently to external cues. Interestingly, our results suggest that this balance has a molecular parallel because CIL and coattraction activate RhoA and Rac1, respectively, two antagonistic Rho GTPases (Figures 7I and 7J). External cues including both repulsive interactions (such as those mediated by Semaphorins and ephrins (Kuriyama and Mayor, 2008), as well as attractive factors (such as VEGF and Sdf1) (McLennan et al., 2010; Theveneau et al., 2010), also play an essential role in directing NC migration. However, NC chemotactic response is highly dependent on local cell interactions, such as CIL, and requires high cell density with almost complete loss of chemotaxis when cells are dispersed into single units (Theveneau et al., 2010). Thus, the intrinsic organization, by coattraction and CIL, of these cells is required for adequate response to extrinsic signals. It is important to clarify that NC cells from other regions of the embryo (such as the enteric NC) or from mammalian and avian embryos are known to adopt other modes of migration. The potential role of the complement in these cells remains to be determined. It may be that the migratory mechanism shown here may not be NC specific but a characteristic of migratory cell clusters.

In summary, here we show that a mechanism that had not hitherto been seen in animal cells, coattraction, may be at the core of collective migration where its role is to maintain the cohesion of cell clusters. This cohesion allows CIL to operate and to generate coherent polarity, imparting directionality to the cell group (Carmona-Fontaine et al., 2008). We have shown that local or social interactions between cells are key to achieve collective migration. We predict that collective migration in many cell types is achieved by a balance between a dispersive force (such as CIL) and an attraction, like coattraction, as we have shown to naturally exist in NC cells and to be sufficient to induce collective migration in hematocytes that otherwise move individually (Figures 7F and 7G). Interestingly, similar balances are widely accepted to explain the swarming behavior in collective animal movements (Buhl et al., 2006; Romanczuk et al., 2009), suggesting that similar strategies for producing collective movement have emerged at different magnitudes and levels of complexity. The coattraction between NC cells is reminiscent of the behavior of *Dictyostelium*, where individual cells release and respond to a chemoattractant to produce a multicellular aggregate. However, coattraction between single NC cells seems to be weak and, therefore, unlikely to lead to aggregation. Slime bacteria, or myxobacteria, also swarm under adverse environmental conditions such as starvation. Lauffenburger et al. (1984) proposed a mechanism for this swarming that is remarkably similar to our own propositions. Based on these examples, it is possible to speculate that there is a limited number of strategies that lead to effective collective migration and that these strategies are repeated over the course of evolution.

A surprising finding of this work is the role of complement proteins in coattraction. An intriguing possibility is that immune cells may exhibit coattraction that is also dependent upon complement. If so, coattraction could have been co-opted by the immune system during vertebrate evolution as a positive feedback mechanism for efficient recruitment of cells to particular sites.

## Experimental Procedures

### General Methods

*Xenopus* embryo microinjections and cell cultures were performed as previously described (Carmona-Fontaine et al., 2008). C3aR and control morpholinos were diluted in pure water to a concentration of 5 mM, and 5 nl per embryo was injected. To analyze NC migration in vitro, NC explants were cultured in plastic or glass dishes coated with fibronectin (Sigma) and filled with Danilchick's solution (DFA). Time-lapse analysis was performed using DIC microscopy or fluorescent microscopy of cells injected with nuclear-RFP/membrane-GFP or membrane-RFP/nuclear-GFP, using a DM5500 Leica compound or a Leica confocal microscope. Chemotaxis assay was performed as described before (Theveneau et al., 2010). Zebrafish strains were maintained and bred according to standard procedures (Westerfield, 2000). Zebrafish manipulation, and time-lapse analysis, was performed as described (Matthews et al., 2008). In situ hybridizations and western blots were performed using standard protocols. The peptides used in this study were synthesized in an Applied Biosystems peptide synthesizer (model 431A; Foster City, CA) using Fmoc-based chemistry (Atherton and Sheppard, 1989). Cloning of C3aR (accession number JN713926) and development of the C3a expression constructs together with more methods and statistical analysis are described in the Supplemental Experimental Procedures. All error estimates correspond to the standard deviation from the mean.

### FRET Analysis and Rac1 Inhibition

Rac1 activity was probed using FRET as previously reported (Carmona-Fontaine et al., 2008; Matthews et al., 2008; Theveneau et al., 2010). Briefly, 100 pg of a DNA vector encoding a Rac1 FRET probe (Itoh et al., 2002) was coinjected with 15 ng of control or C3aR morpholinos. Then, control and C3aR MO NC cultures were treated with either C3a or C3a-DesArg for 20 min. Samples were then fixed, and the FRET efficiency was analyzed. Only single cells were analyzed to avoid the influence of cell-cell contact in Rac1 activity. Rac1 was chemically inhibited using NSC23766 (Tocris) at a concentration of 50 μM.

### Cell Substrate and Cell-Cell Adhesion Assays

To measure the cell substrate adhesion, cell cultures were performed as normal but flipped over after 10, 20, 30, or 40 min. Then the percentage of explants that remained attached was scored. Experiments were done in triplicate. Two experimental approaches were used to analyze differences of cell-cell adhesion. First, cell adhesion was analyzed using a cell-sorting assay as described in Ninomiya et al. (2004) with some minor modifications. The main modification was that cells from differently labeled donors were separately dissociated in Ca^2+^-Mg^2+^-free DFA instead of the 50% Ca^2+^-Mg^2+^-free PBS supplemented with 0.1% BSA (1/2PBSB) previously used.

Then cells were mixed and resuspended with the pipette and then left to reaggregate in agarose-coated wells under gentle agitation. After 1 hr, the aggregates were completely mixed in all conditions. Finally, they were cultured at 14.5°C for 24 hr and then analyzed. Experiments were done in triplicate. A second method that allows quantification of cell adhesion was used. NC cells were cultured as a monolayer on fibronectin. Control and treated NC cells were dissociated, mixed, and seeded on the NC monolayer. After 3–5 min, the dish was flipped over, shaken, and the number of remaining cells was counted. Pictures were taken before and after the dish was flipped over. Cells injected with an N-Cadherin morpholino (Nandadasa et al., 2009) were used as a positive control because they show a clear decrease in cell adhesion as compared to control cells.

### Computer Model

An agent-based model of NC migration was created. Briefly, particles were set to move randomly at constant speed and interact (or not) with neighboring particles. Two types of interactions were modeled: a short-range repulsive interaction aimed to emulate CIL, and a longer-range attractive interaction termed here coattraction. To implement coattraction, cells produce a diffusible attractant whose concentration decays exponentially with distance. At the same time, particles sense this attractant and bias their random movement toward its highest concentration. With these interactions, particles start swarming, and the coattraction of two swarms is achieved. For a more detailed and mechanistic description of the model, please refer to the relevant section in the Supplemental Experimental Procedures.

## Accession Numbers

The sequence for *Xenopus laevis* C3aR has been deposited in GenBank with accession code JN713926.

## Supplemental Information

Document S1. Four Figures and Supplemental Experimental Procedures

Movie S1. NC Can Self-Organize to Generate Collective Directional Migration A labeled NC explant was cultured on lanes with high levels of fibronectin. Note the emergence of directional collective migration even in the absence of attractive and/or repellent cues. Magnification 10×. Sample rate: 1 frame/10 min. Related to Figure 1.

Movie S2. Temporal Evolution of a Model of NC Migration Note that when particles have repulsive interactions (CIL) they are much more efficient than when they do not interact (random), but they lack the collective movement that emerges when mutual attraction (CoA) is also included. Related to Figure 1.

Movie S3. NC Cells Mutually Attract Each Other In Vitro and In Vivo First part: in vitro. Two NC explants migrate toward each other when cultured in close proximity. Second part: in vivo. Ectopic grafts of NC cells (green) join the endogenous stream of NC migration (red) (left). However, if the endogenous NC is removed (right), the grafts disperse radially. Magnification 10×. Sample rate: 1 frame/10 min. Related to Figure 1.

Movie S4. Complement Fragment C3a Is an NC Chemoattractant NC explants cultured close to a source (shown by red and green circles) of control peptide (left), C3a-desArg (center), and C3a (right). Magnification 10×. Sample rate: 1 frame/10 min. Related to Figure 3.

Movie S5. Loss of C3a and C3aR Function Depletes Mutual Attraction First part: Confronted explants of NC treated with a control antibody (left) or with anti-C3a (right). Second part: Confronted labeled explants of NC treated with a control morpholino (left) or C3aR MO (right). Third part: NC coattraction in vivo requires C3aR. Ectopic grafts of NC cells (green) join the endogenous stream of NC migration (red) (left). However, if the receptor C3aR is blocked in the graft (right, green), they are not longer able to join the endogenous NC, but the grafts disperse radially. Magnification 10×. Sample rate: 1 frame/10 min. Related to Figure 4.

Movie S6. Coattraction Is Required for Uniform Response to External Cues Control and C3aR MO-treated NC cells were exposed to the NC chemoattractant SDF1. As expected, control cells respond uniformly to SDF1, but morphant cells have a heterogeneous response. Magnification 10×. Sample rate: 1 frame/10 min. Related to Figure 5.

Movie S7. Mutual Attraction Is Required for NC Collective Migration In Vivo and In Vitro First part: in vivo. NC migration was analyzed in vivo in a graft of GFP-labeled NC cells and treated with a control morpholino (left) or with C3aR MO (right). A mild morphant phenotype is shown. Note that the control graft remains as a cluster, whereas the morphant graft disperses into individual cells. Magnification 5×. Second part: in vitro. NC migration was analyzed ex vivo in labeled NC explants treated with a control morpholino (left) or with C3aR MO (right). Explants were cultured on lanes of high levels of fibronectin flanked by lanes with lower levels (red). Note that control explant remains as a cluster and migrates directionally. On contrary, morphant cells disperse, even to regions of low fibronectin levels. Magnification 10×. Sample rate: 1 frame/10 min. Related to Figure 7.

Movie S8. Coinjection of C3a and C3aR Can Induce Collective Migration in Otherwise Single Cells In *Xenopus* embryos, myeloid cells (green) are formed in the anterior-ventral blood island, from where they disperse as individual cells (left). However, if they are made to express C3a and C3aR, they adopt a collective-like type of migration. Magnification 10×. Sample rate: 1 frame/10 min. Related to Figure 7.
