# Supplementary material for: Complement Fragment C3a Controls Mutual Cell Attraction during Collective Cell Migration
Source: Dev Cell. 2011 Dec 13;21(6):1026–37. doi: 10.1016/j.devcel.2011.10.012 (PMC3272547; doi:10.1016/j.devcel.2011.10.012)
Supplement: Document S1. Four Figures and Supplemental Experimental Procedures [file mmc1.pdf]

Developmental Cell, Volume *21*

Supplemental Information

Complement Fragment C3a Controls Mutual Cell Attraction during Collective Cell Migration

Carlos Carmona-Fontaine, Eric Theveneau, Apostolia Tzekou, Masazumi Tada, Mae Woods, Karen M. Page, Maddy Parsons, John D. Lambris, Roberto Mayor

Supplemental Inventory

Figure S1, Related to Figure 1. NC cells show a significant trend to return to their migratory stream or cluster of origin.
Figure S2, Related to Figure 2. Identification and cloning of C3aR in *Xenopus laevis.*
Figure S3, Related to Figure 4. Single cells are attracted towards cell clusters in a C3a dependent manner.
Figure S4, Related to Figure 7. C3a and C3aR loss of function produce disorganization in NC migration.

Supplemental Experimental Procedures

Supplemental References

Supplemental Figures

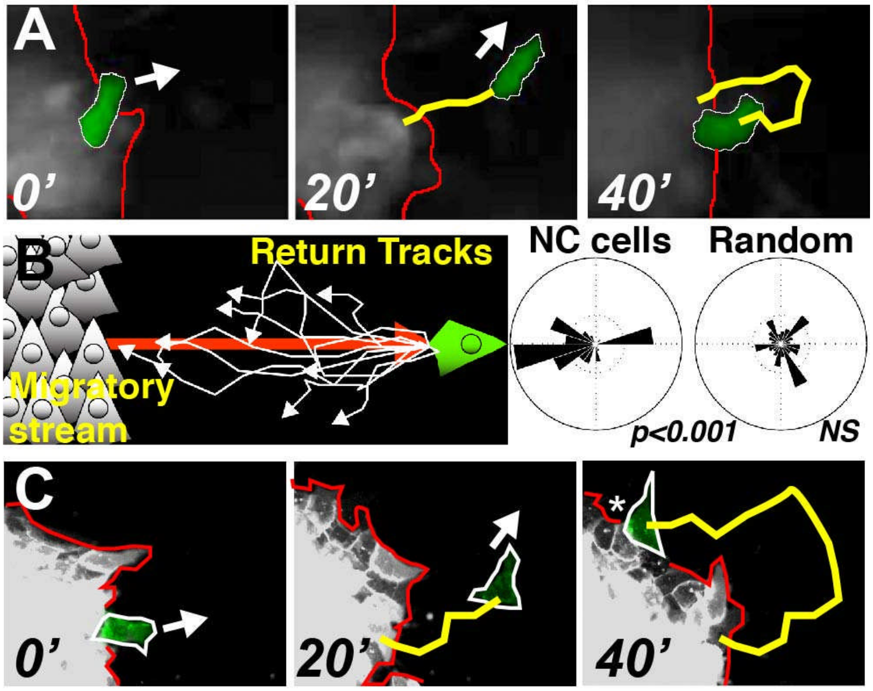

Figure S1. NC cells show a significant trend to return to their migratory stream or cluster of origin. (A) shows that when a cell leaves the migratory stream, it is quickly redirected back to the stream. Track is shown in yellow. Red lines: migratory path. (B) Quantification of redirection behavior shown in A. Cells that move away from the migratory stream were tracked. A vector was created for each track with origin in the stream and final point in the maximum distance that the cell moves away from the stream (red arrow). The tracks for different cells were aligned according to this vector and the angles at each time point were calculated (NC cell graph), and compared with a random distribution (right graph). (C) When a cell leaves the cluster; it quickly goes back to it. Yellow line: track; Red lines: border of migratory area. (Related to Figure 1).

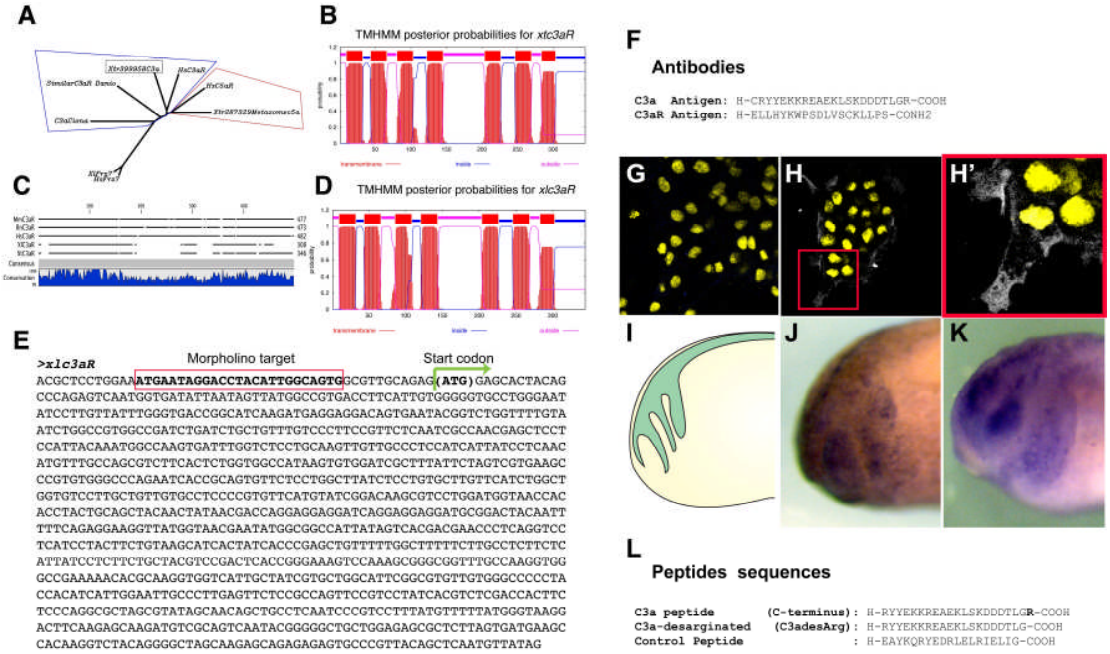

Figure S2. Identification and cloning of C3aR in *Xenopus laevis.* (A, B) A putative C3aR sequence was identified in the genomic sequence of *Xenopus tropicalis* (Xtr899958). (A) This sequence clusters with other C3aR and not with closely related C5aR. (B) The amino acid sequence coded by this gene shows the seven transmembrane regions and the large second extracellular loop typical of C3aR. (C) Using Xtr899958, degenerated primers where designed and together with a RACE, a gene was cloned in *Xenopus laevis.* This gene (called *xlc3aR*) shows a high degree of conservation with other C3aR (C) and the right membrane profile (D). (E) *xlc3aR* sequence. The start codon and the morpholino target region are highlighted. Note that the target region is upstream of the start codon and thus *xlc3aR* mRNA could be used to rescue the morpholino effect. (F) Sequence of the epitopes for C3 and C3aR antibodies. (G-K) C3 and C3aR proteins are present in NC cells. (G,H) Immunohistochemistry of a NC explant and nuclei stained with DAPI (in yellow). (G) No primary antibody to show no background or unspecific staining. (H) NC explant showing staining with C3 antibody. (H’) High magnification of region squared in h. (I) Schematic representation of migrating NC region (green). (J, K) Whole mount immunohistochemistry of C3 and C3aR, respectively. (L) Sequences of the peptides used in this work. (Related to Figure 2).

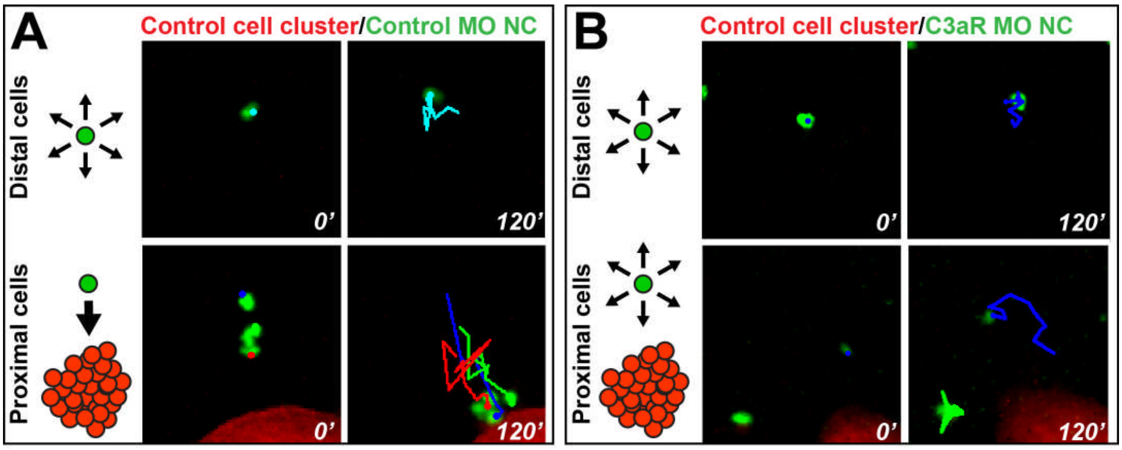

Figure S3. Single cells are attracted towards cell clusters in a C3a dependent manner. (A, B) Control or C3aR MO NC cells labeled with nuclear GFP were dissociated into single cells and then scattered in the vicinities of RFP labeled NC explants. (A) Control cells proximal to the cluster (bottom panels, <300µm) have a trend to migrate towards the cluster, while distant cells are not affected and they move randomly (upper panels). (B) Similar experiments for C3aR morphant NC cells show that these cells exhibited random migration, regardless of their distance to the explant. (Related to Figure 4).

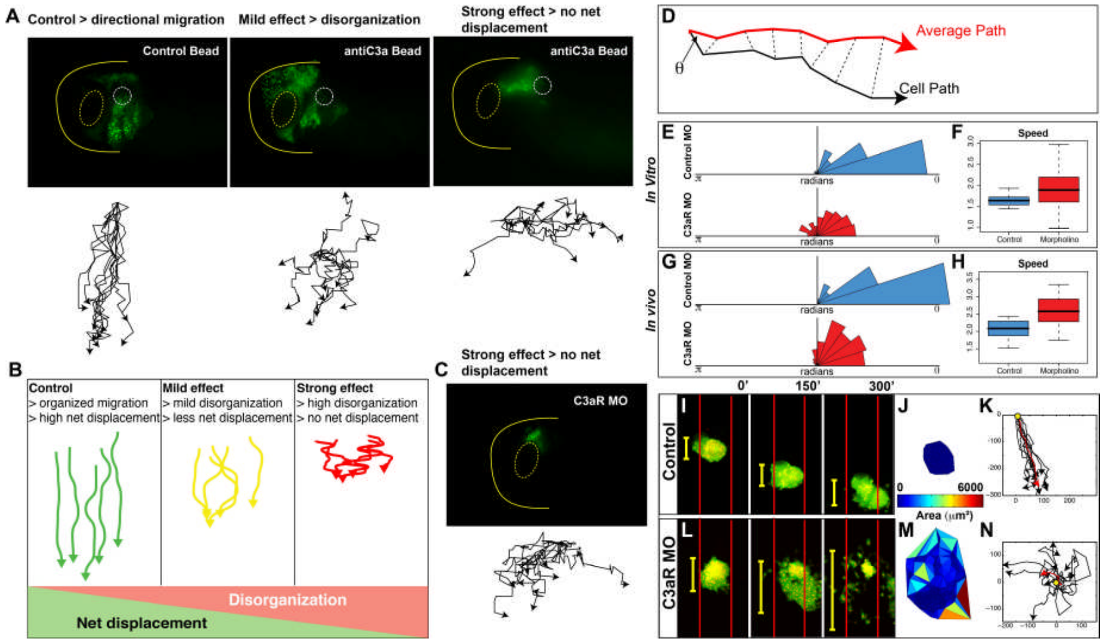

Figure S4. C3a and C3aR loss of function produce disorganization in NC migration. The effect in cell migration was analyzed *in vivo* by direct observation of moving cells. To do this, nuclear-GFP-labeled NC were grafted onto an unlabeled embryo whose endogenous NC cells have been previously removed. (A). Effect of C3a inhibition by treatment with C3a antibody. Top image embryo with labeled NC after 10 hours of migration. Bottom: migratory tracks. Live imaging revealed a similar graded pattern in the phenotype's expressivity. Importantly, it revealed that the observed strong phenotype is not due to a defect in cell motility but a strong disorganization of the migratory stream. (C). Similar to (A) but after C3aR inhibition by C3aR MO treatment. Only strong phenotype is shown as other are extensively analyzed in main Figure 7. (B). Summary of phenotypes. (D) From all the tracks (only one shown, black) an average track is obtained (red). The alignment θ is defined as the angle in between each migratory path and the average path at all time points (here shown only for the first time). θ ranges from 0 to π radians. (E) Rose histograms showing the distribution of θ in cells treated with a control morpholino (blue) or a C3aR morpholino (red) *in vitro*. Note that control cells show a higher frequency of small angles reflecting cell alignment. (F) Box plots showing speed of control (blue) and C3aR morpholino (red) cells showing that their variance is significantly different ($p<0.001$, f-test). (G,H) Similar to (E, F) but for cell migrating *in vivo*. (I) Frames of a movie showing migration of a control NC explant. (J) Dispersion. (K) Migratory tracks including average track (red). (L-N) Similar to (I-K) but for a C3aR morphant explant. (Related to Figure 7).

Supplemental Experimental Procedures

General Methods

Microinjections and cell cultures were performed as previously described (Carmona-Fontaine et al., 2008). Briefly, all injections to target the NC were performed into lateral animal blastomeres at the 16-cell stage (blastomeres V1.2 and D1.2). If injection localization was not important, they were performed into one or both blastomeres at the two-cell stage. Morpholino were diluted in pure water to a concentration of about 3ng/nL and 5nL per embryo was injected. To control the toxicity of the treatment, the same dose of control morpholino was injected. None of these treatments showed significant toxicity. Oligo-morpholinos were designed as recommended by GeneTools. The following morpholinos were used: C3 MO (5’-ACTGGACAATGTGCAAACTTTGAAT-3’), C3aR MO (5’-ATGAATAGGACCTACATTGGCAGTG-3’), and a standard control morpholino from GeneTools.

To analyze NC migration *in vitro*, NC explants were cultured in plastic or glass dishes coated with 10ng/mL of fibronectin (Sigma) and filled with Danilchick's Solution (DFA). Chemotaxis assay was performed as described before (Theveneau et al., 2010) and time-lapse analysis *in vitro* was performed using DIC microscopy or fluorescent microscopy of cells injected with nuclear-RFP/membrane-GFP or membrane-RFP/nuclear-GFP, using a DM5500 Leica compound or a Leica Confocal Microscope.

Zebrafish strains were maintained and bred according to standard procedures (Westerfield, 2000). Zebrafish manipulation, and time-lapse analysis, was performed as described (Matthews et al., 2008). For time-lapse analysis, zebrafish embryos were

orientated laterally to have an orthogonal view to the migration plane. A similar approach was used to do live imaging in *Xenopus* embryos but some modifications were required. First, because of the dramatic extension of the body axis, *Xenopus* embryos were placed in tunnels of agarose. Moreover, NC where taken from a (nuclear-GFP) labeled donor and grafted onto an unlabelled host prior migration. The embryos were positioned laterally. *In situ hybridizations* were performed using standard protocols as described before (Carmona-Fontaine et al., 2007). Western Blots were performed using standard protocols and revealed using chemiluminescent hydrogen peroxide reaction detected in an X-O Graph processor. 10 µg of protein was loaded in all cases.

Patterned-fibronectin surfaces

To make stripes of high levels of fibronectin we used silicon matrices as described in (Vielmetter et al., 1990). Briefly, a matrix with channels of 90µm was filled with a fibronectin (50ng/mL) and fluorescein (for visualization, 1ng/mL) solution. By capillarity, this will form high level of fibronectin stripes, separated by stripes of lower level of fibronectin. This system allowed us to provide a preferential rather than restricted area for NC migration. The stripes are not visible in movies because we used the same channel to visualize the cells nucleus whose fluorescence was much higher. However, at time 0 these stripes were photographed to be able to draw the stripes in movies and figures.

Construction of a C3a expressing vector

To express the C3a peptide without the inclusion of other, potentially active regions of C3 (such as C3b), we designed an Activin-C3a fusion construct using the activin pro-

region. This region has been shown to be an effective tool to ensure the secretion of fusion proteins (Carreira-Barbosa et al., 2009). To do this, first, the entire C3a sequence was cloned by RT-PCR from *Xenopus* stage 30 embryos. The pre-pro region of mouse Activin BetaA subunit (GenBank accession number: X69619) including the processing site **RRRR** was then fused to the mature C3a, thus creating pCS2-Act-C3a.

Cloning of *xlc3aR*

A *Xenopus laevis* version of C3aR has not been previously reported so it needed to be cloned. To do this, a putative homologue was identified in the *Xenopus tropicalis* genome. We identified a sequence, Xtr899958, as *xtC3aR* through sequence conservation. It clustered with mammalian versions of C3aR but not with versions of the highly related C5aR. Moreover, a sequence profile revealed the expected seven putative transmembrane domains plus a large second extracellular loop characteristic of this gene (Fig S1a-e). We used this sequence to design degenerated primers to amplify the *X. laevis* version from cDNA obtained from total mRNA expression at embryonic stage 30. The primers used were TTGAAGTCYYTGCCCATRAARACRTA (fwd) and GTGGCYGAYYTNATYTGYTTRTC (rev). A digoxigenin-labeled antisense probe was synthesized from this sequence to determinate the expression pattern of the gene via *in situ* hybridization. After corroborating it was expressed in the NC, sequence-specific primers were designed and a RACE was performed using the 5’/3’ RACE Kit, 2n Generation (ROCHE) following manufacturer’s instructions with an additional nested amplification step. With this, the whole coding sequence of *xlC3aR* and about 200bp from the 5’ region were obtained. The latter was used to design an antisense

oligomorpholino (Fig S1e). The sequence was conserved with other versions of C3aR, especially to the *X. tropicalis* one. As expected, the sequence profile of xlC3aR also revealed seven putative transmembrane domains and the large second extracellular loop. The identity of the gene was also confirmed functionally as *xlc3aR* is expressed in migratory NC and is required for their response to C3a (see main text). The sequence of C3aR is publically available in the NCBI genebank database (Accession number JN713926).

Peptides Synthesis and antibody production

The peptides used in this study were synthesized in an Applied Biosystem peptide synthesizer (model 431A, Foster City, CA) using Fmoc-based chemistry (Atherton and Sheppard, 1989). All peptides were HPLC purified to 95% purity, and the identity of the peptides was confirmed by laser desorption mass spectrometry (MALDI). Antibodies against synthetic peptides were made in rabbits by subcutaneous injections of the synthetic peptides coupled to keyhole limpet hemocyanin by glutaraldehyde (Reichlin, 1980) and m-Maleimidobenzoyl-N-hydroxysuccinimide ester (peptides containing free cysteine) (Kitagawa and Aikawa, 1976). The rabbit immunoglobulin was fractionated from other serum proteins by ammonium sulphate precipitation and the specific antibody was purified by affinity chromatography on the corresponding peptide column. See table S1 for the sequences of peptides and antigens used to develop antibodies.

Peptide and antibody treatments in embryos and cell cultures

Treated cell cultures were performed in the same way as untreated ones but peptides, chemicals or antibodies were directly diluted in the DFA solution. See table S2 for used working concentrations. In the *in vitro* chemotactic assay, beads soaked in different solutions were used. Briefly, heparin-acrylic beads (Sigma) were washed in PBS and then soaked in a peptide/antibody solution overnight at 4ºC or for a few hours at room temperature. Then beads were placed next to the fibronectin surface and fixed with non-toxic silicon grease. Immediately after, NC cultures were performed and the explants were placed at close proximity (<500µm) (Theveneau et al., 2010). To graft beads *in vivo* a similar procedure was used but Affi-Gel Blue beads (Bio-Rad, 100-200 mesh) were used, as they are easier to handle in embryos. The type of bead did not affect the results. Occasionally -when very high concentrations of peptides or antibodies were required- beads were dehydrated in ethanol, air-dried and re-hydrated with the stock protein solution. Then beads were grafted near the neural crest region before the onset of migration.

Peptide and Antigen Sequences

C3a peptide (C-terminus representing the active site of C3a): H-RYYEKKREAEKLSKDDDTLGRCOOH

C3a-DesArg peptide: -RYYEKKREAEKLSKDDDTLG-COOH

Control Peptide: H-EAYKQRYEDRLELRIELIG-COOH

Working Concentration of Reagents

C3a: 0.7µM; C3aDesArg: 0.7µM; Control Peptide: 0.7µM; anti-C3a: 1µg/mL; antiC3aR: 1µg/mL; SB290157 [C3aR antagonist (Calbiochem). Stock solution diluted in DMSO. Final DMSO concentration in working solution]: 0.2%, 20µM.

Quantifications and Statistical Analysis

Statistical analysis was done in Matlab (Mathworks), R (r-project.org) and/or excel (Microsoft). Cell tracking was performed using the manual tracker plug-in of ImageJ and then processed in Matlab. Persistence was calculated dividing the distance of the end point of a cell from its origin by the total length of its path. All directional statistics were using custom-made Matlab scripts implementing the modifications by Moore to the Rayleigh's test (Moore, 1980) as described before (Carmona-Fontaine et al., 2008). To calculate the Chemotactic Index (CI), the coordinate system was rotated to have the y-axis passing through the source of the chemoattractant at the top and the cells at the bottom. Then the CI will correspond to the cosine of the angle between the displacement of the cell and the y-axis (Kay et al., 2008). A CI value of 1 (angle value 0) means that the cell is directly going towards the chemoattractant. To calculate the average path, the Cartesian positions of the tracked cells were averaged at each time point. With this, the angle between the displacement of each cell and the direction of the average displacement can be obtained for each time step. This angle reflects the alignment between cells as lower values will indicate aligned cells and higher values will indicate that cells are departing from the general directional trend. To do this, θ was calculated using, $\theta_i = \arccos\left(\frac{c_i \bullet c_a}{|c_i||c_a|}\right)$ where, c_i is the displacement vector for the cell *i* and c_a is the

average displacement vector at a particular time point, $c_i \bullet c_a$ is the dot product between those two vectors and $|c| = \sqrt{c \bullet c}$.

Delaunay Triangulation to measure cell dispersion

When trying to measure the dispersion of groups of cells several problems arise. Because it is sometimes difficult to control exactly the initial number of cells, especially with primary cultures, simply measuring the final surface covered for the explant can be very misleading. Also, the ratio between the initial and the end explant surface can be misleading because the explant may dissociate in a heterogeneous way and thus, differences may be averaged out. To overcome these difficulties, we devised a way to measure dispersion based in more local differences rather in global differences. It consists in the simple idea that in situations where you have more dispersion, you will have bigger areas in between neighbors. To determinate the nearest neighbors, Matlab/Qhull Delaunay triangulation algorithm (Barber et al., 1996) were used in 2D. For a set **P** of points on a plane, this triangulation requires that no point in **P** is inside the circumscribed circle of any triangle of the triangulation. In the following figure, a visual explanation of how the algorithm works is provided for clarity. Once the triangulation was obtained, the areas of the triangles were calculated in Matlab. Finally, triangles were depicted in a color-coded fashion where the color is proportional to their areas. To compare different treatments or to analyze repetitions of the same experiment areas were statistically compared. The natural logarithm of the area values was used to have the data normally distributed. Colors represent different arbitrary treatments. Then, the logarithm of the areas was analyzed, using one-way ANOVA and depicted in box plots.

Analysis of myeloid cell migration and induction of collective migration.

First embryos were injected with a combination of actC3a and C3aR mRNA plus fluorescein (FDX) as a lineage marker or with FDX alone. At stage 15, the anteriorventral mesoderm of labeled embryos was removed and transplanted onto unlabeled embryos. This is the region that contains the myeloid cell precursors (Costa et al., 2008). Then the migration of these cells was analyzed using an epifluorescence microscope.

Computer model of Neural Crest migration.

Outline

A model of neural crest (NC) migration was created using realistic parameters and according the biological scenario: the NC cell population is initially localized at the dorsal midline from where it delaminates and migrates ventrally. Although NC cells migrate in a 3D space, in the model we have assumed a 2D space. This assumption is based in the following observations. Z-stack analysis performed in preliminary experiments established how deep the NC migrates in the zebrafish embryo. After 6 to 8 h time lapse of 20 somites embryos, we found that cephalic NC migrate between 500-800μm in the anterior-posterior axis, between 40-60μm in the dorso-ventral axis and between 7 and 9μm in the periphery-center axis. In consequence, it is possible to assume that most of the cell migration is performed in two dimensions, while the third dimension (the Z axis when the embryo has a lateral orientation) can be neglected. In addition, it was observed that migration of cephalic NC is highly directional from dorsal to ventral, and it is known that they follow specific pathways, which are flanked by negative signals.

We constructed an agent-based model where particles (cells) are initially localized at the top (dorsal) of a surface (a 2D lattice) laterally limited by regions where the cells cannot migrate (as in the embryo). At the bottom (ventral), there is a region where cells stop their migration (target). For modeling coattraction of two cell clusters, two NC subpopulations were initialized at close proximity but not adjacent.

In the onset of the model, the position of the *N* number of particles and the boundary conditions are set. Typically, the velocities and particle positions are initialized as a normally distributed cluster with random velocities. When running the model, cells were prompted to move as random walkers. In one or two-dimensional manifolds, it can be proven that as the number of steps in a random walk approaches infinity, the probability of reaching the origin (and thus any other position) equals the unity. This means that in theory, all cells in the model will eventually reach the target. The question addressed by the model is whether it is possible to reduce the number of steps required to reach the target by including cell-cell interactions.

Explicitly, each particle has an internal clock set to a random value, ranging from 0 to its maximum value (*Step*), at the model onset. Moreover, the particles are set to have different interaction rules. We focused on three cases: no interactions (or random), repulsive interactions (CIL) and repulsive plus attractive interactions (CIL+CoA). These interactions are given by different radii as shown in panel A.

The *Step* parameter was defined as the number of iterations a cell will continue moving in the same direction before changing direction randomly. This parameter allows the migration of a cell in a non-random manner for a small fixed number of iterations, and therefore, it helps to distinguish the effect on migration produced by the collision

during contact inhibition from the random migration at the end of the *Step*. If CIL is considered, at any time that the cell collides, *Step* is reset, thus the cell will continue for a *Step* number of iterations in the new direction given by the collision (unless it collides again).

If particles display CIL when they collide, apart from resetting their internal clock, they align for one iteration and then bounce from each other with some noise given by a random angle ranging from 0 to θ radians in the next one. Cells without CIL are not affected by the collision. The region of collision (rCIL) is given by the particle (cell) diameter (panel A).

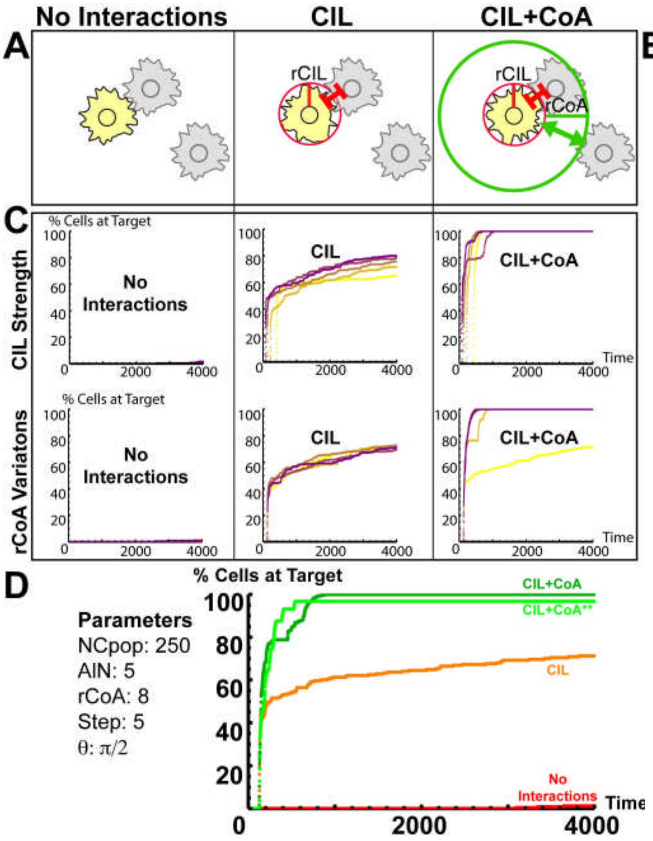

Graphic display of the cell-cell interactions included in the three modeled situations. Note rCIL < rCoA. **B.** When coattraction is considered, each cell secretes attractant which diffuses with an exponential distribution. Thus, the maximum value of attractant can occur at the center of the group. Dotted line: estimation of the attractant concentration after adding the production of each cell (green line). **C.** Parameter sweep for the three types of models. Efficiency is measured as % of cells at the target over time. Top: Different values of CIL strength. (Yellow = 1, Purple = 10). Bottom: Different values of rCoA (Yellow = 1, Purple = 10). Neither of the parameters changes the qualitative outcome of the model. **D.** Summary of all treatments. Note that CoA is always more efficient. CIL+CoA** controls that the cells in the target are not influencing the ones moving.

For the inclusion of the coattraction (CoA), an interaction radius rCoA (bigger than the CIL radius) was defined for each particle (Panel A, note rCoA>rCIL). Particles will sense particles within this radius but only when the internal clock (given by *Step*) has the maximum value. If these conditions are met, the particle will move towards the center of mass of the particle population +/- some random noise. A second model was developed based on diffusion of the co-attractant (see below) which gives similar results.

Agent-based model algorithm.

```
Setup initial conditions
       - Set boundary conditions -> migration area surrounded by non-migratory
       areas and a target area at the very bottom
       - Set particles ->  seed NCpop number of cells at random locations near
       the top of the migratory areas
       - Set persistence to random values (between 0 and Step) for different
       particles
       - Set time counter tc = 0

Migration iterations
       - Ask particles Check differentiation
       - Ask particles Bounce
       - Ask particles Move
       - Set time counter tc = tc+1

Routines
       Check differentiation
              If location ahead = target area,
                     - Set color = orange
                     - Stop being active particle
       Bounce
              If location ahead = non-migration area,
                     - Turn in 180 – (2x incidence angle) degrees.
                     - Move forward
       Move
              If color = orange
                     - Stop
              Else
                     If persistence < 1  and CoA is OFF
                            - Change direction randomly
                            - Move forward
                            - Set persistence = Step
                     If persistence < 1  and CoA is ON
                            - Detect center of mass of particles within CoA
                            radius
                            - Turn in that direction + noise
                            - Move forward
                            - Set persistence = Step
                     If persistence > 1
                            If CIL is OFF
                                   - Set persistence = persistence – 1
                                   - Move forward
                            If CIL is ON and other particles within particle size
                            radius
```

```
			- Average direction of particles within
			particle size radius
			- Turn in that direction
			- Set persistence = Step
			- Move forward
			- Turn to a random direction
		Else
			- Set persistence = persistence – 1
			- Move forward
```

Model of diffusion and profile of the co-attractant

The mutual attractant concentration is modeled by the function φ

$$\varphi(x,y) = A\sum_{j=1}^{N} e^{-\frac{1}{d}\sqrt{\left(\left(x-x_j\right)^2+\left(y-y_j\right)^2\right)}}$$

where A is the maximum of the function describing the distributed secretion of mutual attractant originating from each particle, d is the diffusion length of mutual attractant and (x_j, y_j) are the coordinates of a particle j.

This equation gives an exponential distribution of the attractant around each particle. When particles are in a cluster, because of the addition of gradients emitted from individual cells, the attractant is distributed so as its highest concentration is at the center of the cluster (Panel B).

A circular sensing radius *rCoA* is defined and the value of φ is calculated, for simplicity, at eight equally spaced points on the circumference and upon a concentration response update the particle is reoriented to the point with the highest value of φ.

This model is very robust and predicts that two groups of particles with CIL and CoA will always meet with the condition that the tail of attractant from one cluster is be perceived by at least one marginal cell of the other explant.

Robustness to parameter sweep.

The presented model is very robust, as its qualitative predictions do not depend on the actual values of its parameters. For example, the strength of CIL, that establishes how long cells are aligned after collision, did not change the behaviour of particles, even when it was set to one (Top panels in C). For simplicity, all the following simulations were performed with strength =1 so cells do not change their speed during CIL. Similarly, the length of the attraction radius rCoA was also not a determinant of the behaviour of the group (Bottom panels in C). The only exception was that when rCoA was set to zero (meaning that there was no attraction). In this case, the particles behaved as in the condition with CIL only (compare middle panel with yellow line in bottom right panel in C). This controls that the addition of CoA is not altering any behaviour of the model other than attraction. Values of all key parameters were sweeped and none changed the qualitative outcome of the model.

The plot in Panel D summarises the results obtained in the model over time. Particles with no interaction showed very poor efficiency (measured as the percentage of particles that reach the bottom region) whereas particles with CIL showed a dramatic increase in the migration efficiency. However, the addition of coattraction makes the particles not only more efficient but also makes them move cohesively. In these models, particles at the target can still influence the behaviour of motile particles. To control this effect, this influence was eliminated and the behaviour of the particles is virtually unchanged (condition CIL+CoA** , light green). The output of these simulations may be better appreciated on Supplementary Movie S2.

This demonstrates that coattraction may be an essential cell interaction in collective migration. The fact that the model is so robust, suggests that coattraction may not be a rare phenomenon because it does not require specific parameter conditions. It also indicates that we are laying a set of minimal conditions that suffice for the emergence of collective migration.

Supplementary References

Atherton, E., and Sheppard, R.C. (1989). Solid phase peptide synthesis: a practical approach, Vol 53 (IRL Press).

Barber, C.B., Dobkin, D.P., and Huhdanpaa, H.T. (1996). The Quickhull algorithm for convex hulls. ACM Trans on Mathematical Software *22*.

Carmona-Fontaine, C., Acuna, G., Ellwanger, K., Niehrs, C., and Mayor, R. (2007). Neural crests are actively precluded from the anterior neural fold by a novel inhibitory mechanism dependent on Dickkopf1 secreted by the prechordal mesoderm. Dev Biol *309*, 208-221.

Carmona-Fontaine, C., Matthews, H.K., Kuriyama, S., Moreno, M., Dunn, G.A., Parsons, M., Stern, C.D., and Mayor, R. (2008). Contact inhibition of locomotion in vivo controls neural crest directional migration. Nature *456*, 957-961.

Carreira-Barbosa, F., Kajita, M., Morel, V., Wada, H., Okamoto, H., Martinez Arias, A., Fujita, Y., Wilson, S.W., and Tada, M. (2009). Flamingo regulates epiboly and convergence/extension movements through cell cohesive and signalling functions during zebrafish gastrulation. Development *136*, 383-392.

Costa, R.M., Soto, X., Chen, Y., Zorn, A.M., and Amaya, E. (2008). spib is required for primitive myeloid development in Xenopus. Blood *112*, 2287-2296.

Grashoff, C., Hoffman, B.D., Brenner, M.D., Zhou, R., Parsons, M., Yang, M.T., McLean, M.A., Sligar, S.G., Chen, C.S., Ha, T., *et al.* (2010). Measuring mechanical tension across vinculin reveals regulation of focal adhesion dynamics. Nature *466*, 263-266.

Itoh, R.E., Kurokawa, K., Ohba, Y., Yoshizaki, H., Mochizuki, N., and Matsuda, M. (2002). Activation of rac and cdc42 video imaged by fluorescent resonance energy transfer-based single-molecule probes in the membrane of living cells. Mol Cell Biol *22*, 6582-6591.

Kay, R.R., Langridge, P., Traynor, D., and Hoeller, O. (2008). Changing directions in the study of chemotaxis. Nat Rev Mol Cell Biol *9*, 455-463.

Kitagawa, T., and Aikawa, T. (1976). Enzyme coupled immunoassay of insulin using a novel coupling reagent. J Biochem *79*, 233-236.

Matthews, H.K., Marchant, L., Carmona-Fontaine, C., Kuriyama, S., Larrain, J., Holt, M.R., Parsons, M., and Mayor, R. (2008). Directional migration of neural crest cells in vivo is regulated by Syndecan-4/Rac1 and non-canonical Wnt signaling/RhoA. Development *135*, 1771-1780.

Moore, B. (1980). A modification of the Rayleigh test for vector data. Biometrika *67*, 175-180.

Nandadasa, S., Tao, Q., Menon, N.R., Heasman, J., and Wylie, C. (2009). N- and E-cadherins in Xenopus are specifically required in the neural and non-neural ectoderm, respectively, for F-actin assembly and morphogenetic movements. Development *136*, 1327-1338.

Ninomiya, H., Elinson, R.P., and Winklbauer, R. (2004). Antero-posterior tissue polarity links mesoderm convergent extension to axial patterning. Nature *430*, 364-367.

Reichlin, M. (1980). Use of glutaraldehyde as a coupling agent for proteins and peptides (New York, Academic Press).

Theveneau, E., Marchant, L., Kuriyama, S., Gull, M., Moepps, B., Parsons, M., and Mayor, R. (2010). Collective chemotaxis requires contact-dependent cell polarity. Dev Cell *19*, 39-53.
Vielmetter, J., Stolze, B., Bonhoeffer, F., and Stuermer, C.A. (1990). In vitro assay to test differential substrate affinities of growing axons and migratory cells. Exp Brain Res *81*, 283-287.
Westerfield, M. (2000). The zebrafish book. A guide for the laboratory use of zebrafish (Danio rerio). 4th edn (University of Oregon Press, Eugene).
