# Supplementary figures and images for: Complement Fragment C3a Controls Mutual Cell Attraction during Collective Cell Migration
Source: Dev Cell. 2011 Dec 13;21(6):1026–37. doi: 10.1016/j.devcel.2011.10.012 (PMC3272547; doi:10.1016/j.devcel.2011.10.012)

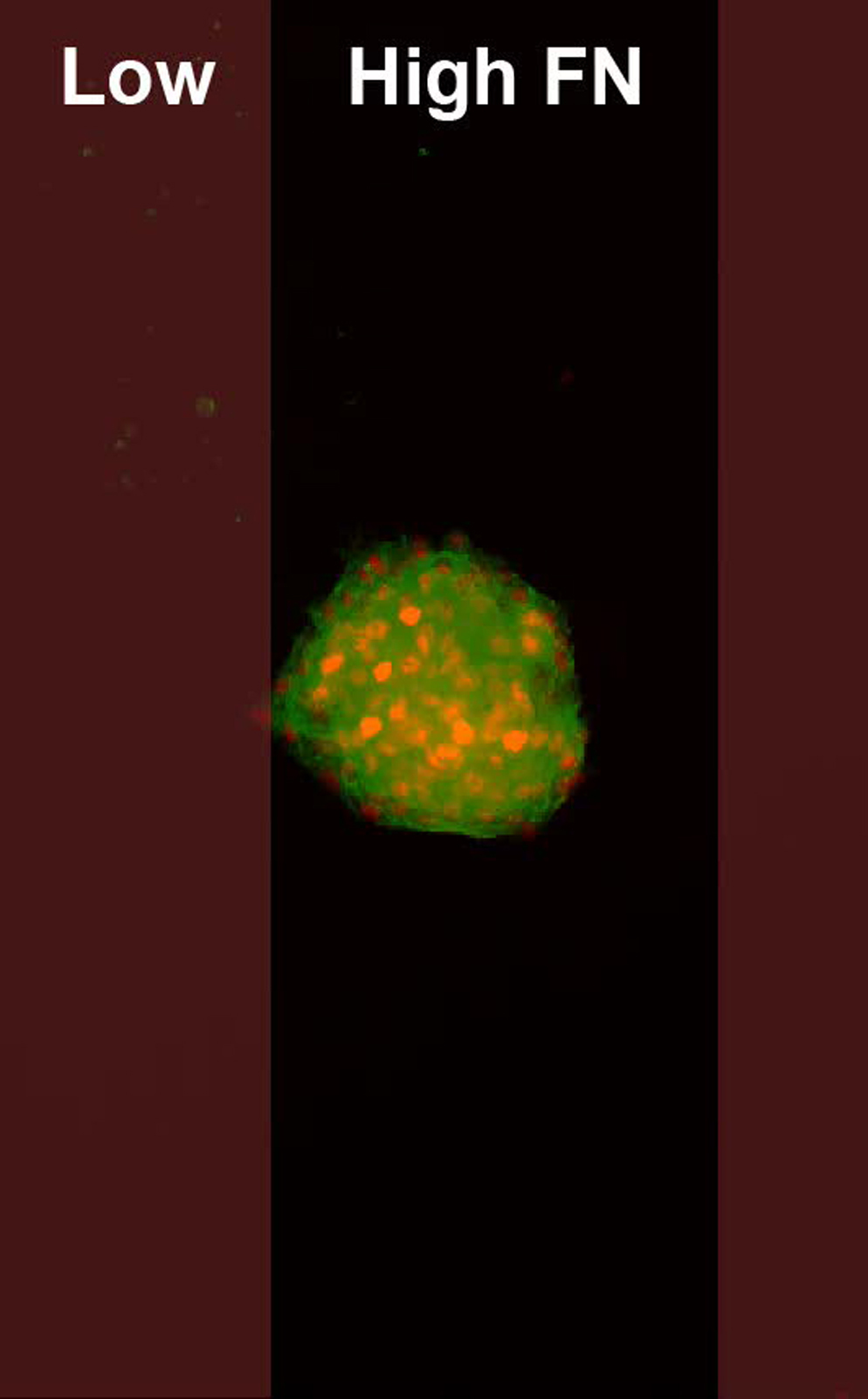

Supplement: Movie S1. NC Can Self-Organize to Generate Collective Directional Migration — A labeled NC explant was cultured on lanes with high levels of fibronectin. Note the emergence of directional collective migration even in the absence of attractive and/or repellent cues. Magnification 10×. Sample rate: 1 frame/10 min. Related to Figure 1. [file mmc2.jpg]

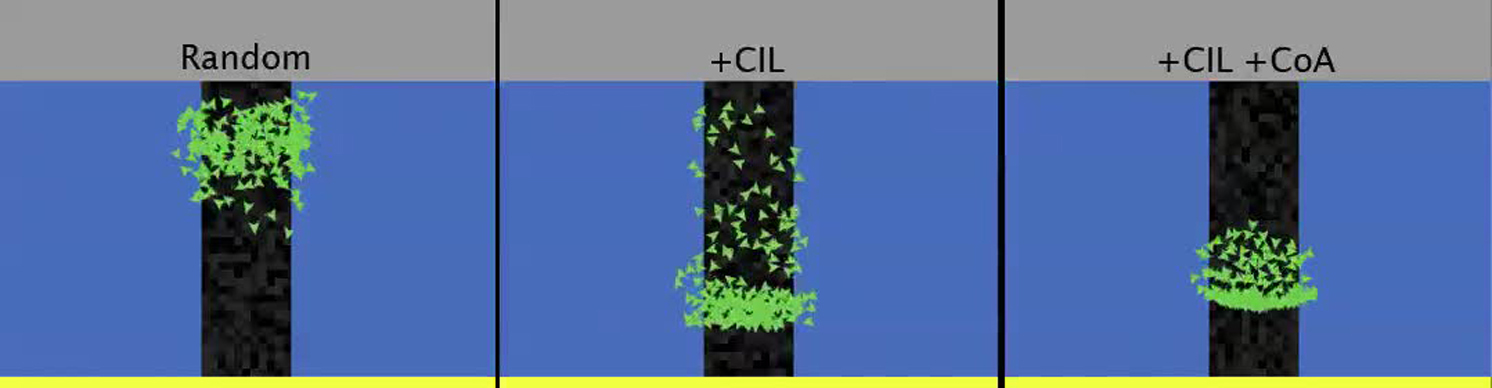

Supplement: Movie S2. Temporal Evolution of a Model of NC Migration — Note that when particles have repulsive interactions (CIL) they are much more efficient than when they do not interact (random), but they lack the collective movement that emerges when mutual attraction (CoA) is also included. Related to Figure 1. [file mmc3.jpg]

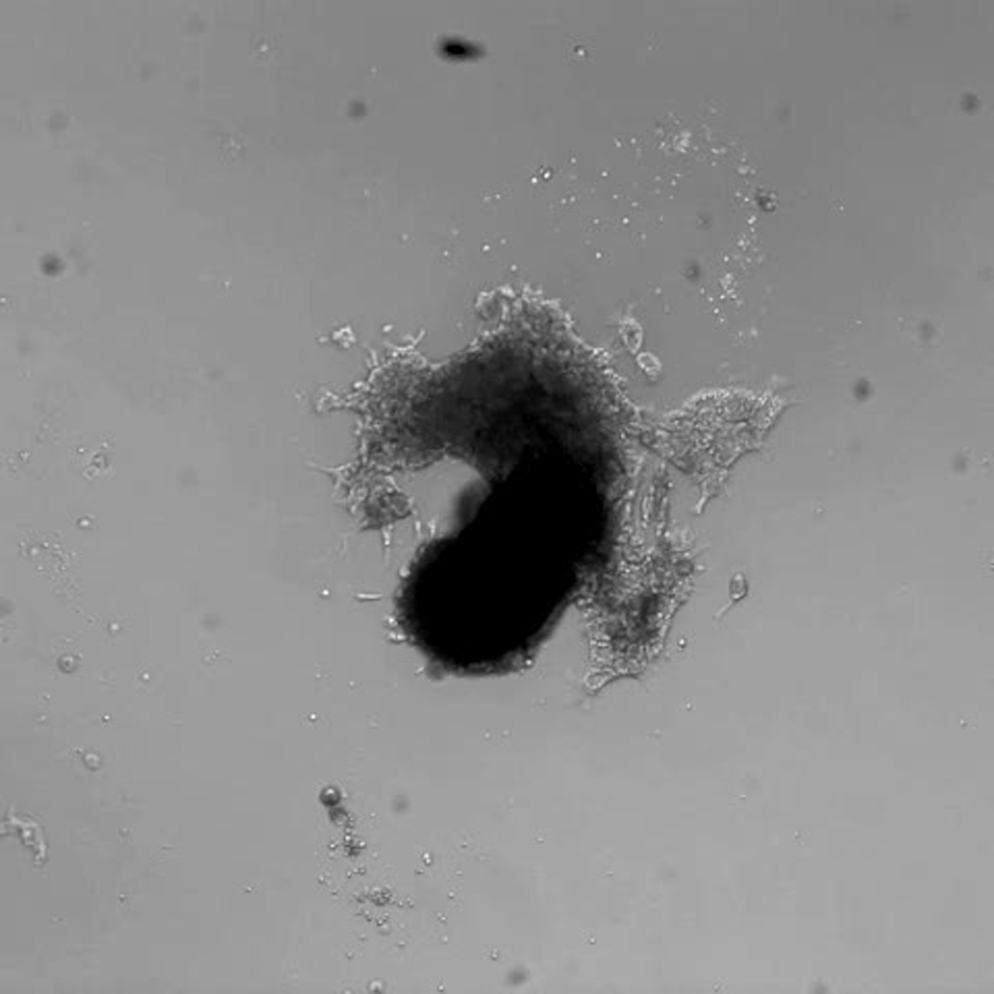

Supplement: Movie S3. NC Cells Mutually Attract Each Other In Vitro and In Vivo — First part: in vitro. Two NC explants migrate toward each other when cultured in close proximity. Second part: in vivo. Ectopic grafts of NC cells (green) join the endogenous stream of NC migration (red) (left). However, if the endogenous NC is removed (right), the grafts disperse radially. Magnification 10×. Sample rate: 1 frame/10 min. Related to Figure 1. [file mmc4.jpg]

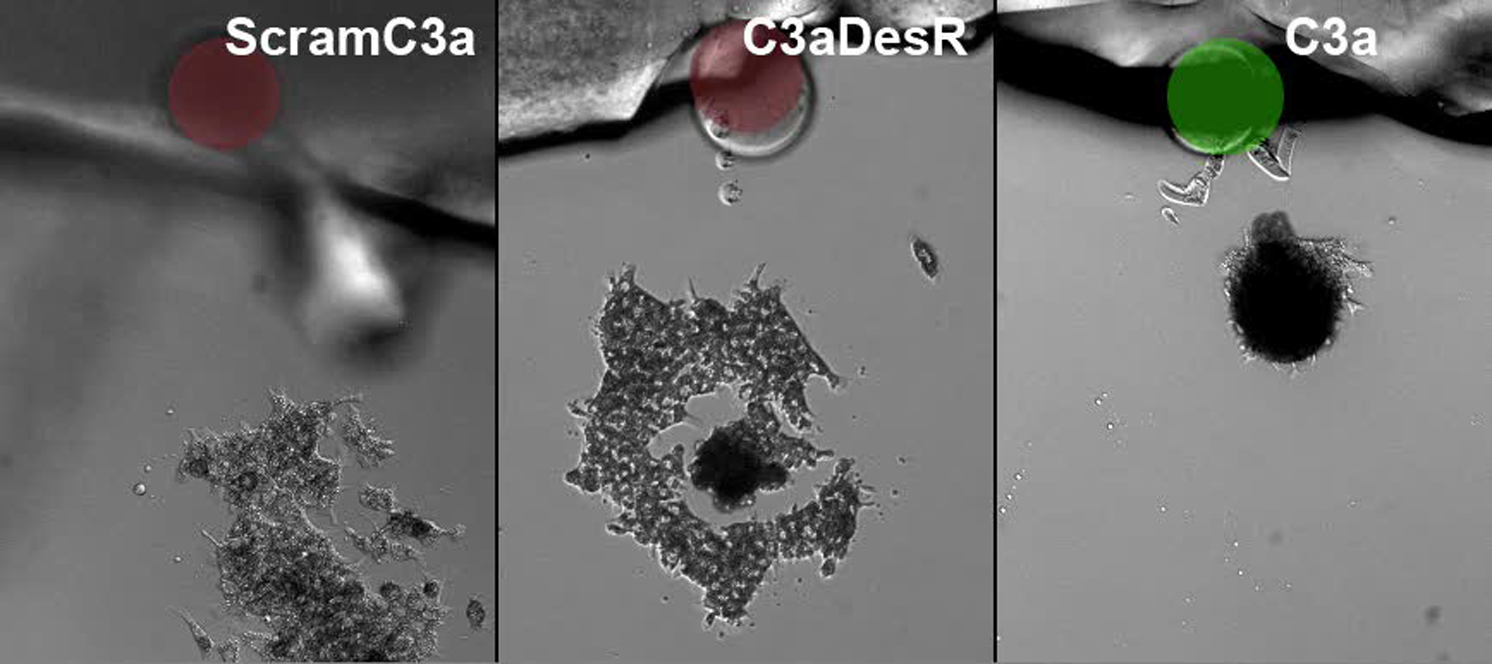

Supplement: Movie S4. Complement Fragment C3a Is an NC Chemoattractant — NC explants cultured close to a source (shown by red and green circles) of control peptide (left), C3a-desArg (center), and C3a (right). Magnification 10×. Sample rate: 1 frame/10 min. Related to Figure 3. [file mmc5.jpg]

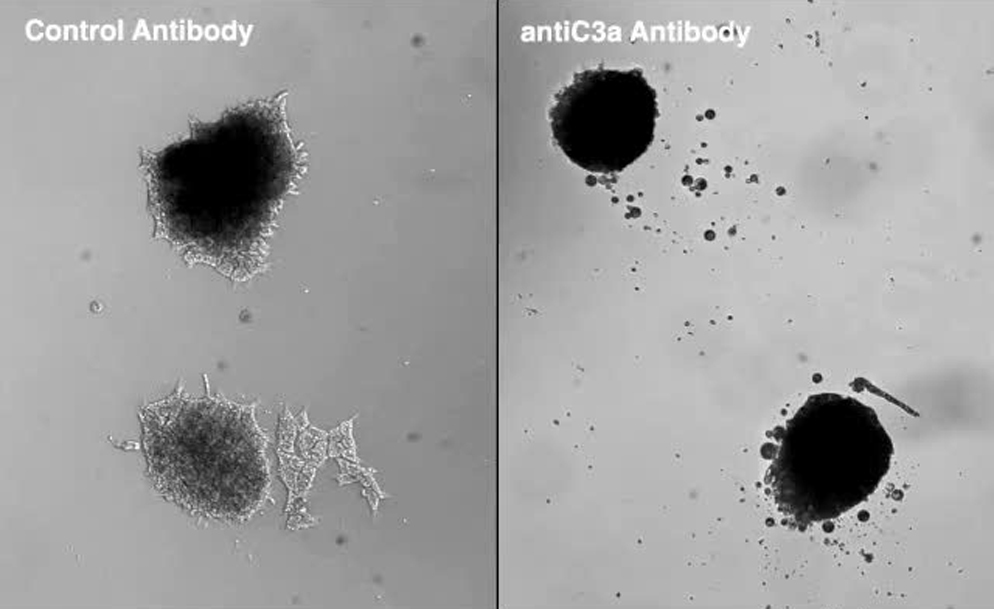

Supplement: Movie S5. Loss of C3a and C3aR Function Depletes Mutual Attraction — First part: Confronted explants of NC treated with a control antibody (left) or with anti-C3a (right). Second part: Confronted labeled explants of NC treated with a control morpholino (left) or C3aR MO (right). Third part: NC coattraction in vivo requires C3aR. Ectopic grafts of NC cells (green) join the endogenous stream of NC migration (red) (left). However, if the receptor C3aR is blocked in the graft (right, green), they are not longer able to join the endogenous NC, but the grafts disperse radially. Magnification 10×. Sample rate: 1 frame/10 min. Related to Figure 4. [file mmc6.jpg]

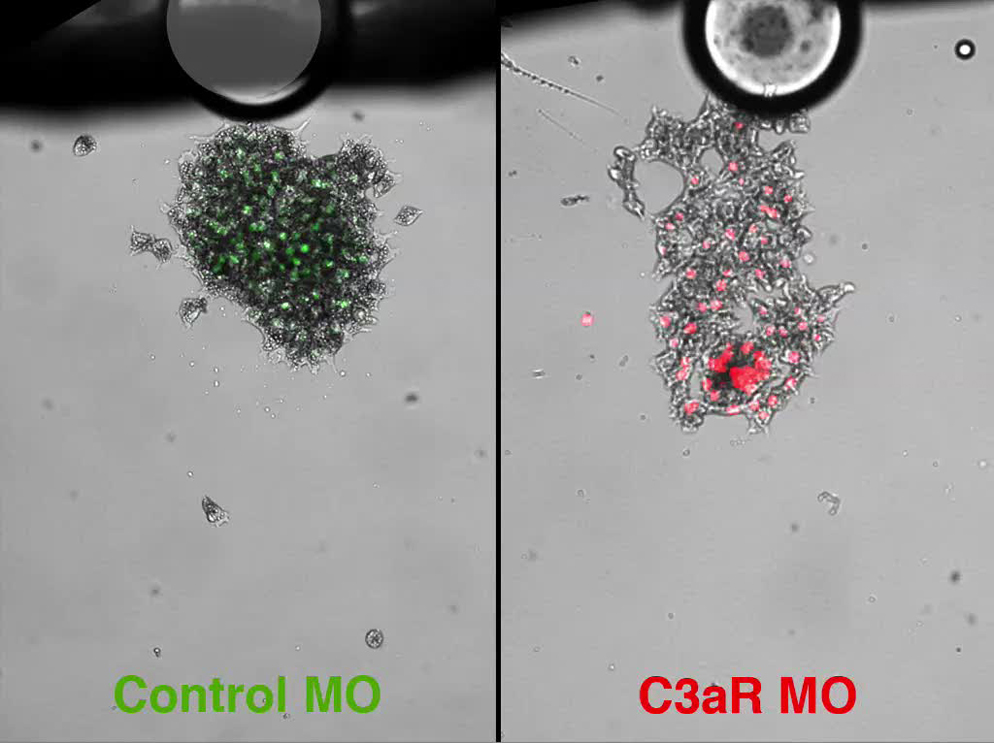

Supplement: Movie S6. Coattraction Is Required for Uniform Response to External Cues — Control and C3aR MO-treated NC cells were exposed to the NC chemoattractant SDF1. As expected, control cells respond uniformly to SDF1, but morphant cells have a heterogeneous response. Magnification 10×. Sample rate: 1 frame/10 min. Related to Figure 5. [file mmc7.jpg]

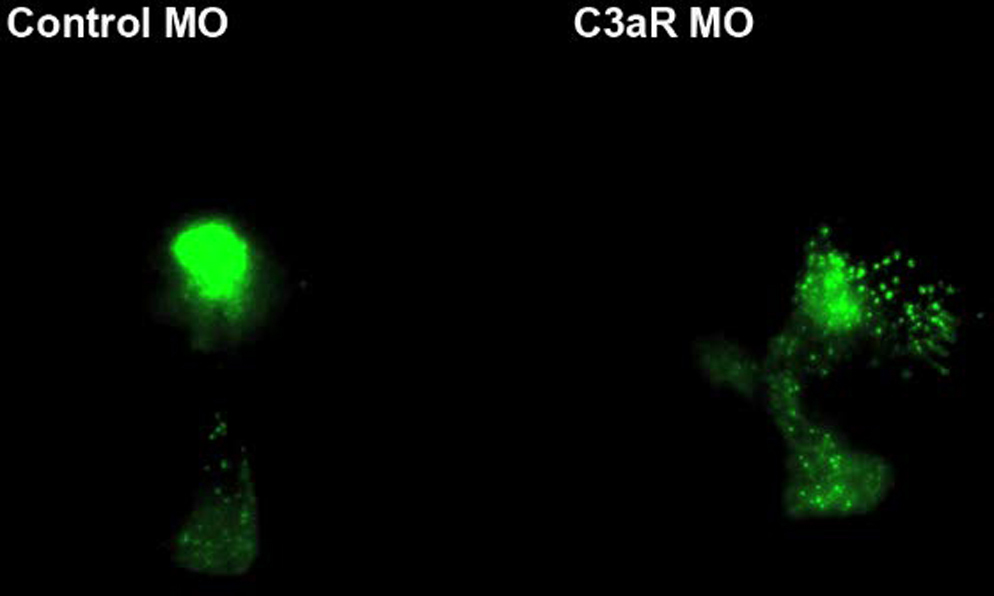

Supplement: Movie S7. Mutual Attraction Is Required for NC Collective Migration In Vivo and In Vitro — First part: in vivo. NC migration was analyzed in vivo in a graft of GFP-labeled NC cells and treated with a control morpholino (left) or with C3aR MO (right). A mild morphant phenotype is shown. Note that the control graft remains as a cluster, whereas the morphant graft disperses into individual cells. Magnification 5×. Second part: in vitro. NC migration was analyzed ex vivo in labeled NC explants treated with a control morpholino (left) or with C3aR MO (right). Explants were cultured on lanes of high levels of fibronectin flanked by lanes with lower levels (red). Note that control explant remains as a cluster and migrates directionally. On contrary, morphant cells disperse, even to regions of low fibronectin levels. Magnification 10×. Sample rate: 1 frame/10 min. Related to Figure 7. [file mmc8.jpg]

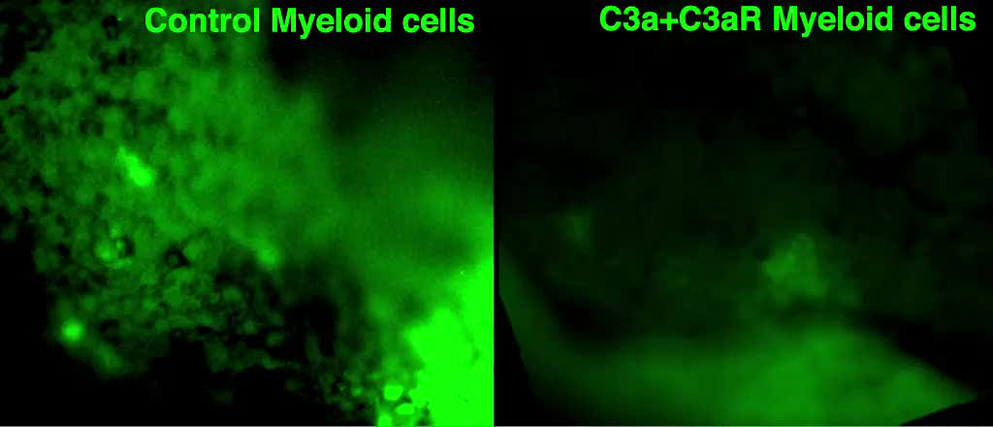

Supplement: Movie S8. Coinjection of C3a and C3aR Can Induce Collective Migration in Otherwise Single Cells — In Xenopus embryos, myeloid cells (green) are formed in the anterior-ventral blood island, from where they disperse as individual cells (left). However, if they are made to express C3a and C3aR, they adopt a collective-like type of migration. Magnification 10×. Sample rate: 1 frame/10 min. Related to Figure 7. [file mmc9.jpg]
